# The current status, challenges and prospects of using biomass energy in Ethiopia

**DOI:** 10.1186/s13068-021-02060-3

**Published:** 2021-10-26

**Authors:** Natei Ermias Benti, Gamachis Sakata Gurmesa, Tegenu Argaw, Abreham Berta Aneseyee, Solomon Gunta, Gashaw Beyene Kassahun, Genene Shiferaw Aga, Ashenafi Abebe Asfaw

**Affiliations:** 1 Department of Physics, College of Natural and Computational Sciences, Wolaita Sodo University, P.O. Box 138, Wolaita Sodo, Ethiopia; 2 Center for Environmental Science, College of Natural and Computational Sciences, Addis Ababa University, P. O. Box 1176, Addis Ababa, Ethiopia; 3 Department of Physics, College of Natural and Computational Sciences, Addis Ababa University, P. O. Box 1176, Addis Ababa, Ethiopia; 4 Department of Physics, Collage of Natural and Computational Sciences, Wollo University, Dessie, Ethiopia; 5 Department of Physics, College of Natural and Computational Sciences, Mettu University, P. O. Box 382, Mettu, Ethiopia; 6 Department of Natural Resource Management, College of Agriculture and Natural Resource Management, Wolkite University, P. O. Box 07, Wolkite, Ethiopia; 7 Applied Physics Program, Adama Science and Technology University, P. O. Box 188, Adama, Ethiopia; 8 Department of Physics, College of Natural and Computational Sciences, Debre Birhan University, P. O. Box 445, Debre Birhan, Ethiopia

**Keywords:** Biomass resource potential, Challenges, Ethiopia, Opportunities, Renewable energy

## Abstract

Despite enormous challenges in accessing sustainable energy supplies and advanced energy technologies, Ethiopia has one of the world's fastest growing economies. The development of renewable energy technology and the building of a green legacy in the country are being prioritized. The total installed capacity for electricity generation in Ethiopia is 4324.3 MW as on October, 2018. Renewable energy accounts for 96.5% of total generation; however, despite the county's enormous biomass energy potential, only 0.58% of power is generated using biomass. Ethiopia has surplus woody biomass, crop residue and animal dung resources which comprise about 141.8 million metric tons of biomass availability per year. At present the exploited potential is about 71.9 million metric tons per year. This review paper provides an in-depth assessment of Ethiopia's biomass energy availability, potential, challenges, and prospects. The findings show that, despite Ethiopia's vast biomass resource potential, the current use of modern energy from biomass is still limited. As a result, this study supports the use of biomass-based alternative energy sources without having a negative impact on the socioeconomic system or jeopardizing food security or the environment. This finding also shows the challenges, opportunities and possible solutions to tackle the problem to expand alternative energy sources. The most effective techniques for producing and utilizing alternate energy sources were also explored. Moreover, some perspectives are given based on the challenges of using efficient energy production and sustainable uses of biomass energy in Ethiopia as it could be also implemented in other developing countries. We believe that the information in this review will shed light on the current and future prospects of biomass energy deployment in Ethiopia.

## Background

The global energy demand is increasing and is expected to continue to increase with predicted population growth and the expansion of energy-dissipative economic activities in the coming decades [1]. Despite significant advances in renewable energy technology, fossil fuels still control the bulk of the energy market [2], which are directly linked to greenhouse gas (GHG) emissions and climate change. However, the trend of primary energy sources indicates that renewable energy will be the fastest-growing energy source over the next two decades [3]. Biomass accounts for more than one-third of primary energy. Concerns about global climate change, acid rain, air pollution from the use of fossil fuels, and advancements in biomass technology have revived interest in biomass energy as a renewable and sustainable energy sources. The use of biomass, along with other renewable energy sources, can help to meet the world's growing energy demand.

Biomass energy, or bioenergy, is created when biomass is converted into electricity, heat, power, or transportation fuels. Because trees and plants can be grown, harvested, and re-grown in a short period of time, biomass is a renewable energy resource. Furthermore, this process generates residues, wastes, and gases continuously [4]. For basic cooking and lighting, more than 80% of the sub-Saharan African (SSA) population relies on solid biomass, such as firewood, charcoal, agricultural by-products, and animal waste [5]. These biomass fuels are burned in unventilated kitchens using smoky and inefficient conventional stoves with poor combustion, resulting in a significant concentration of hazardous pollutants, primarily carbon monoxide and particulate matter, as well as nitrogen oxides and polyaromatic hydrocarbons [6]. Furthermore, exposure to indoor air pollution increases the incidence of acute lower respiratory infections (ALRI) in children and adult chronic obstructive pulmonary disease (COPD) in adults [6, 7].

In 2010, bioenergy accounted for 12% of the world's total final energy consumption, with 9% coming from traditional sources and 3% from modern bioenergy [8]. Therefore, to meet international goals to double the global share of renewables by 2030, a rapid increase in the use of modern biomass is necessary. Solid biomass is the most common source of energy in SSA, accounting for around 70% of the continent's total energy consumption. Approximately 280 million tons of oil equivalents of solid biomass are now utilized in SSA, accounting for 90% of household energy [5, 6]. Almost all of this is wood, straw, charcoal, or dried animal and human waste, which is largely used as cooking fuel. Of the approximately 915 million inhabitants in SSA in 2012, an estimated 730 million (about 80%) have no access to clean cooking facilities [5, 6]. While biomass offers many benefits for the worldwide mix of renewable energy, it is inefficiently exploited in most SSA nations, resulting in a significant degradation of forest resources and a slew of negative consequences for the climate, human health, and social well-being. As a result, utilizing biomass to deliver modern energy services to the world's poor in a sustainable and efficient manner remains critical for community development.

Ethiopia has one of Africa's fastest growing economies, but it has one of the world's poorest access to modern energy supplies. The majority of Ethiopia's population lives in rural areas and is heavily reliant on agriculture; the primary source of energy for this rural population is biomass (biomass of wood, solid, and agricultural wastes) (Table 1), accounting for approximately 87% of total energy supply [9]. Nevertheless, there are significant differences in the current energy systems in rural and urban areas. Almost all rural households rely on traditional biomass for cooking and baking, whereas approximately 90% of urban populations rely on electricity for lighting. Ethiopia has enormous biomass energy potential, but it is not being utilized efficiently and effectively. Ethiopia's estimated exploitable biomass potential and currently exploited biomass potential are 141.8 and 70.9 million tons per year [5], respectively (see Table 1).

**Table 1:** Biomass energy potential of Ethiopia [5]

Resource	Exploitable potential (million tons per year)	Currently exploited (million tons per year)
Woody biomass	74	60
Crop residue	38	4.9
Dung	29.8	7
Grand total	141.8	71.9

Despite its heavy reliance on traditional energy sources, the country is gradually transitioning away from non-renewable energy sources and toward a clean and renewable energy supply. Because of the fast-growing economy and flourishing infrastructures, energy demand is currently increasing at an alarming rate [9]. Therefore, finding an alternative energy source to overcome the issues associated with traditional biomass energy sources could be advocated at its best.

This review paper provides an in-depth assessment of biomass energy sources in Ethiopia, along with remarks on its availability, potential, opportunity, and challenges. The review also discusses the current and future prospect of biomass energy deployment in Ethiopia and their conversion processes are also presented briefly.

## Energy context in Ethiopia

### Access to energy

**Fig. 1:**
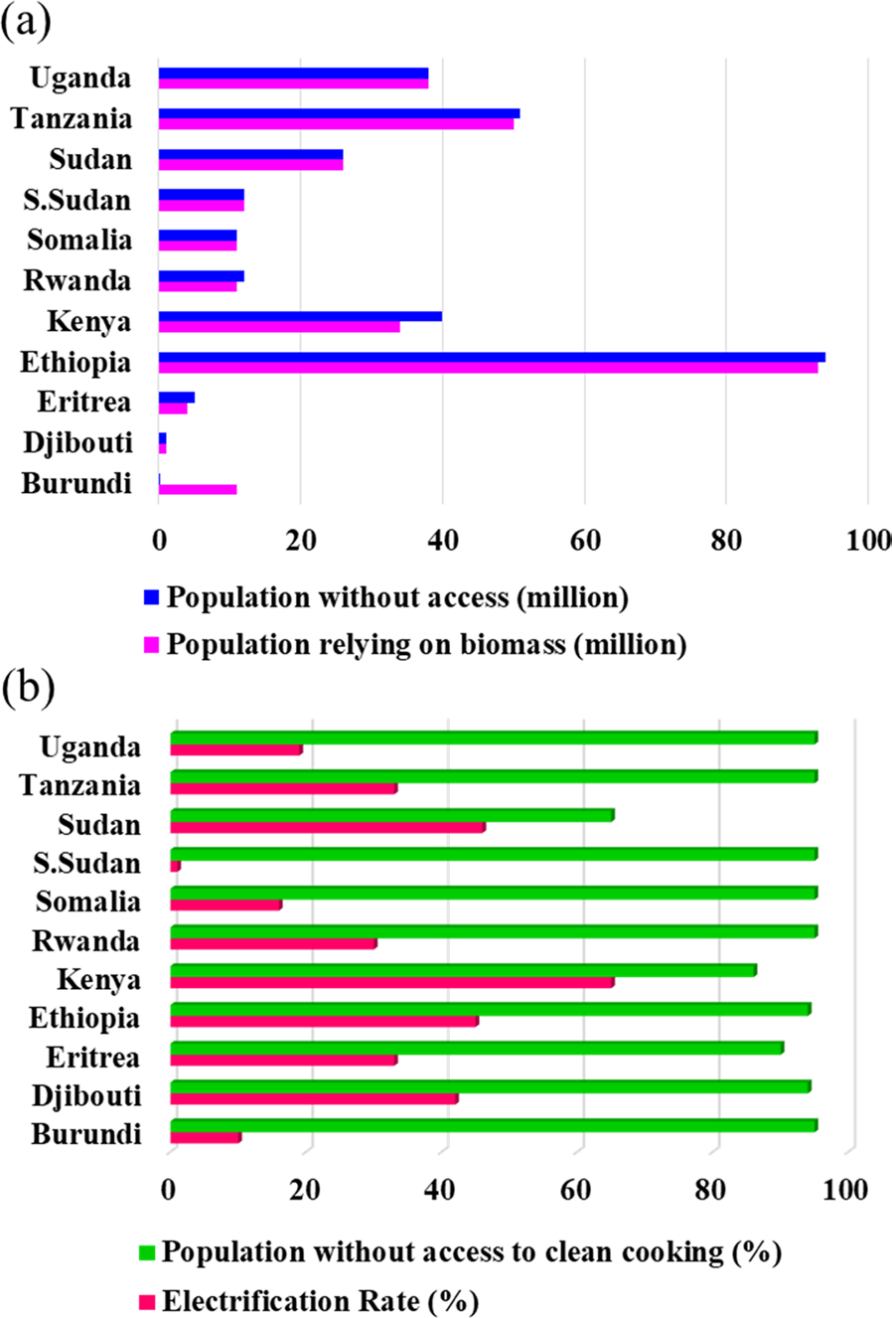
**a** Populations relying on biomass and those who live without access; **b** rate of electrification and populations without access of clean cooking in East Africa as of 2016 [10]

Nearly 1.06 billion people in the world do not have access to electricity [10] and 2.5 billion people still use traditional energy to meet their cooking requirements. Moreover, its accessibility varies widely across regions and the situation is dismal in the least developed countries (LDCs) and SSA. According to WEO 2017, the rate of electrification in SSA has nearly tripled since 2012, compared to the rate between 2000 and 2012. East Africa, in particular, has made significant progress, with the number of people without access dropping by 14% since 2012 (see Fig. 1) [10]. Despite this turn-around, 590 million people roughly 57% of the population remain without access in SSA, making it the largest concentration of people in the world without electricity access as efforts have often struggled to keep pace with population growth. Over 80% of those without electricity live in rural areas, where the electrification rate is less than 25%, compared with 71% in urban areas [10].

**Fig. 2:**
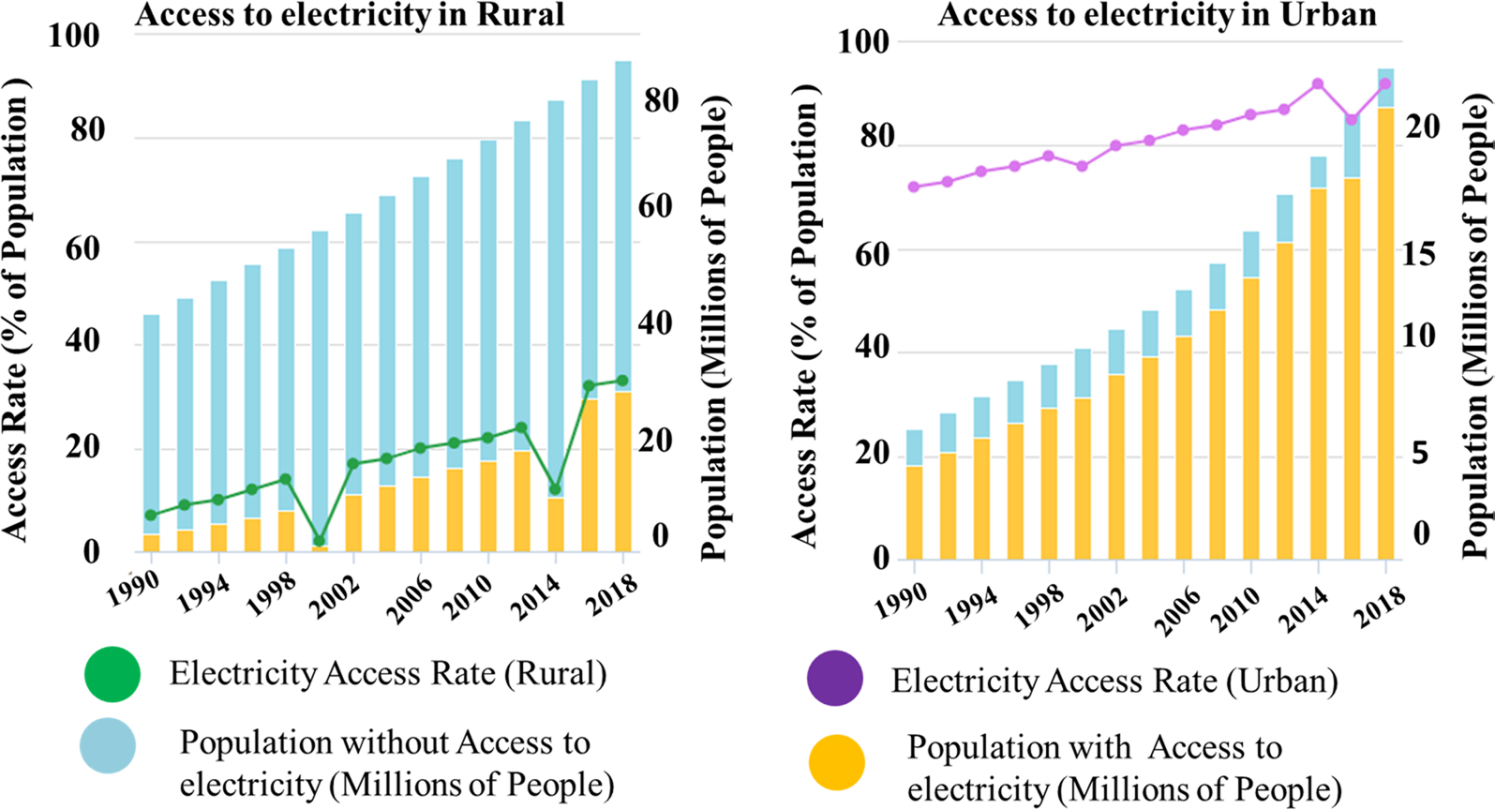
Urban and rural access to electricity in Ethiopia (1990–2018) [11]

In 2016, approximately 45% and only 6% of Ethiopia's total population had access to electricity and clean cooking, respectively (Fig. 1b) [10]. About 85% of Ethiopia's urban population has access to public electricity. This figure is only 29% for the rural population (Fig. 2). In Ethiopia, approximately 93 million people rely on solid biomass for cooking [11]. Over 90% of domestic energy needs are met by biomass, which contributes to deforestation, soil nutrient loss, and organic matter loss. In any case, Ethiopia is one of the countries that places a high value on biomass (Fig. 1) [10].

### Overall energy production and consumption

**Fig. 3:**
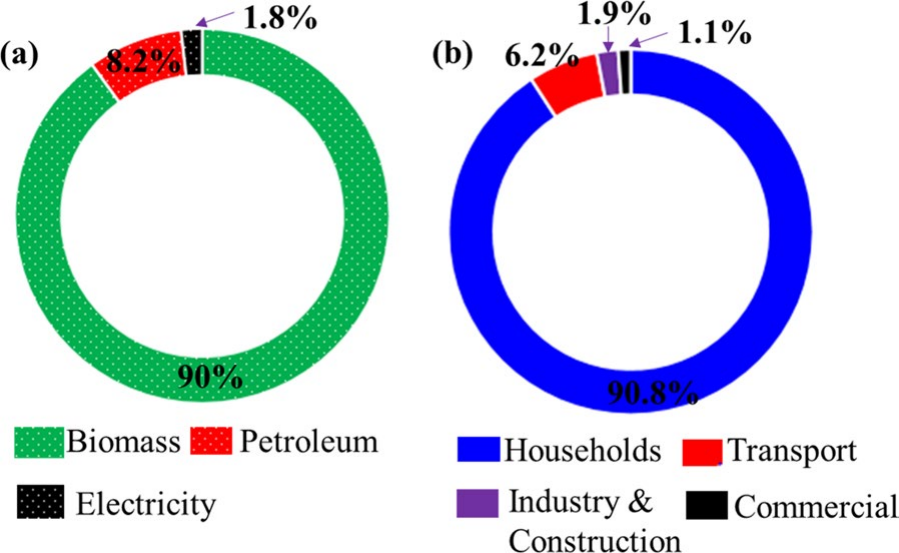
**a** Energy supply in Ethiopia by type. **b** Energy consumption by sector

There are three main sources of energy in Ethiopia. These are biomass, petroleum, and electricity of which, only petroleum products have been imported. From 37,357 ktoe of total energy supply in 2014, the share of biomass was 33,645 ktoe (90%) and energy supplied from Petroleum products and Electricity is 3712 ktoe which accounts for 10% of the total (3047 ktoe from petroleum product and 665 ktoe is from electricity, accounting for 8.2% and 1.8%, respectively) [12] (Fig. 3a). In the same year, the energy consumption of Ethiopia was 35,192 ktoe from which, the share of biomass was 90% (31,699 ktoe) and 8.5% (2973 ktoe) and 1.5% (520 ktoe) was fulfilled by petroleum products and electricity, respectively [12].

Households, transport, industry and construction and commercial sectors are identified as the more energy-consuming sectors in Ethiopia. The final energy consumption of Ethiopia was grown to 35,583 ktoe in 2014. Households and transportation sectors were the two largest energy-consuming sectors, accounting for 32,323 ktoe (90.8%) and 2213 ktoe (6.2%) of energy consumed, respectively. They were followed by industry and construction 656 ktoe (1.9%) and commercial 391 ktoe (1.1%) in 2014 (Fig. 3b) [12]. In Ethiopia, like many developing countries, non-commercial biomass plays a big role in energy supply, especially in the household sector. The transport, agriculture, commercial and industrial sectors rely mainly on commercial energy, especially petroleum fuels and electricity.

In general, the energy profile of Ethiopia can broadly be defined by biomass energy specifically the traditional use of biomass for cooking. Most of the biomass energy is used for cooking in the household sector. Being dependent on the traditional use of biomass, the energy utilization of the country is inefficient and unsustainable. The largest portion of biomass energy is lost as waste energy to the environment due to the use of very low energy efficiency traditional cooking technology; consequently, only a very small portion of it becomes useful energy.

### Electricity generation

**Fig. 4:**
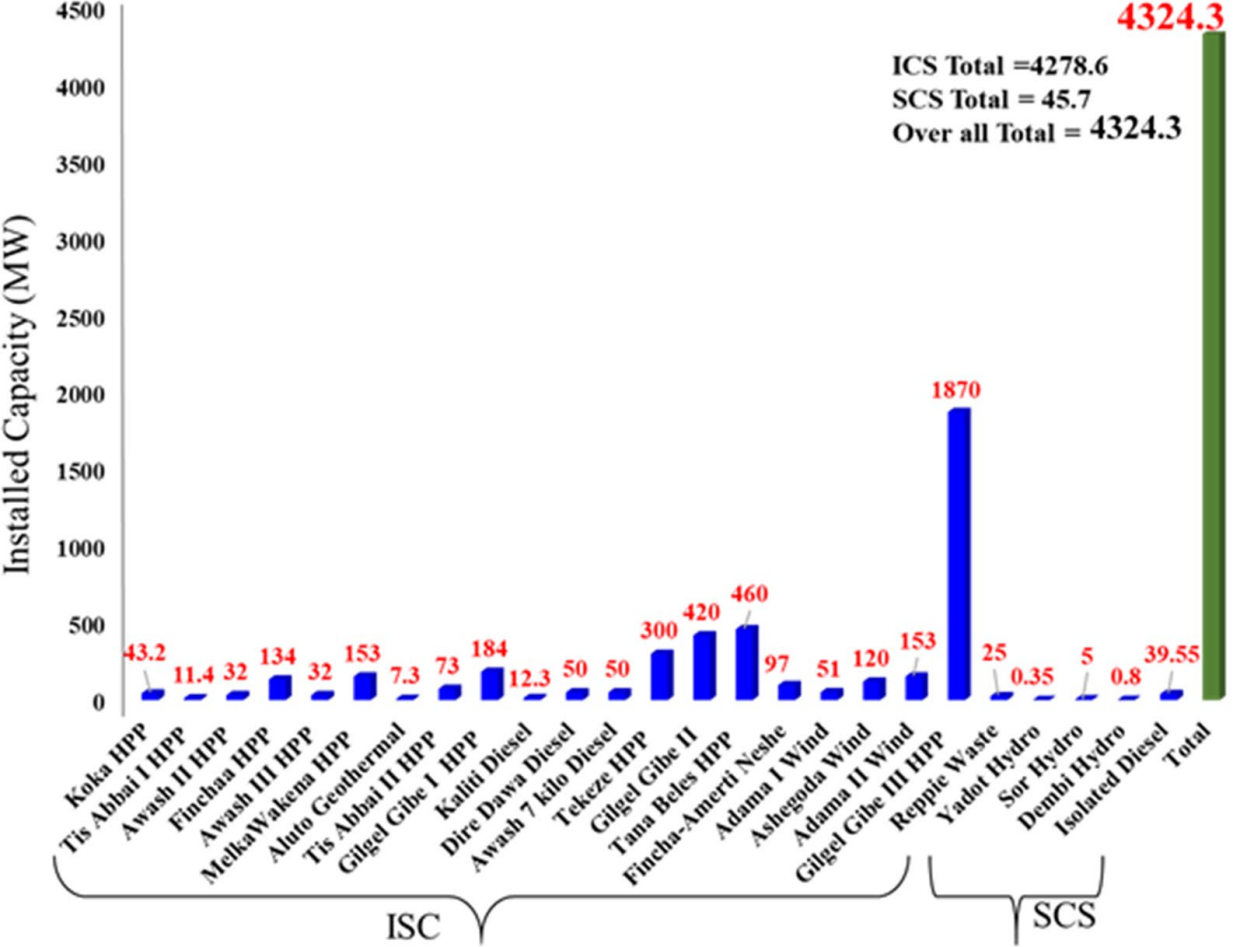
Existing power plants installed capacity (MW) to the national grid of Ethiopia

Ethiopia is endowed with renewable and sustainable energy sources. These include hydropower and, to a lesser extent, wind, geothermal and solar as well as biomass. The approximate potential for hydropower is around 45 gigawatts (GW), for wind is 10 GW and for geothermal is 5 GW, and solar irradiation ranges from 4.5 kilowatt-hours (kWh)/m^2^/day to 7.5 kWh/m^2^/day [13]. Only a small amount of the renewable energy potential is harnessed today. Grid electricity is the main source of modern energy in Ethiopia. Today electricity in the country is produced from hydro, geothermal, wind, biomass (Reppie Waste-to-Energy) and diesel. The total installed electric power generation capacity as of October 2018 was 4324.3 MW (Fig. 4), comprising a mix of hydropower, wind generation, diesel, geothermal and waste-to-energy from municipal solid wastes. The interconnected system (ICS) and self-connected system (SCS) are the two power supply systems in the country. ICS consists of 13 hydropower plants (3810 MW), 3 wind farms (324 MW), Reppie Waste-to-Energy (25 MW) and 3 diesel generators (112.3 MW) (see Fig. 4). The diesel generators in this system served as an emergency power plant, which is mainly used to mitigate the effect of fluctuations in hydropower due to poor rainfall during dry seasons. Diesel power plants rely on expensive imported petroleum fuel, which leads to a high cost of electricity. The high cost has hurt economic activities in the agriculture, manufacturing and transport sectors. The other system is SCS, which consists of diesel generating units and three small hydropower plants that operate in remote areas. Generation in this system is mainly by diesel power plants having an aggregate capacity of 39.55.7 MW by the end of 2016. The contribution from the small hydropower plants is only 6.15 MW (Yadot, Sor and Dembi hydro, 0.35, 5 and 0.8 MW, respectively) despite the availability of many small rivers and waterfalls that could be used for electricity generation to supply many off-grid rural areas in Ethiopia.

**Fig. 5:**
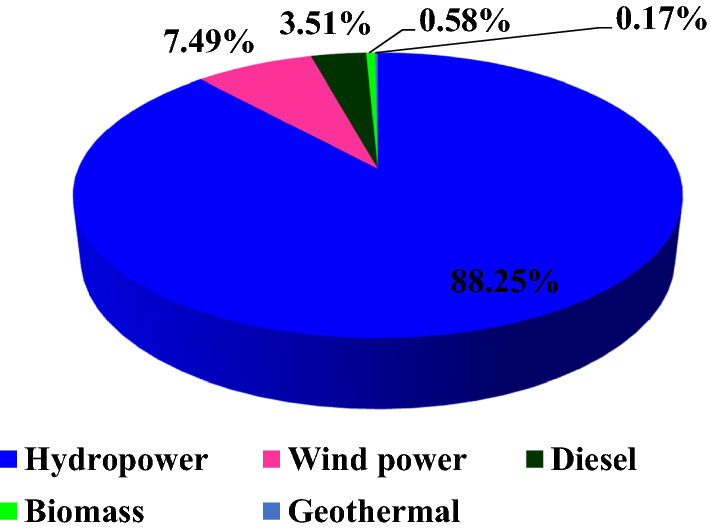
Ethiopian electric power generation by source

Looking at the share of total installed capacity of the country's power plants, only 3.51% of the total generated electricity comes from diesel; the rest is from clean renewable energy resources with 88.25% from hydropower plant, 7.49% from wind power, 0.58% from biomass (Reppie Waste-to-Energy) and 0.17% from a geothermal plant (Fig. 5), which makes Ethiopia’s electricity among the most sustainable in the world.

### Petroleum supply and consumption

**Fig. 6:**
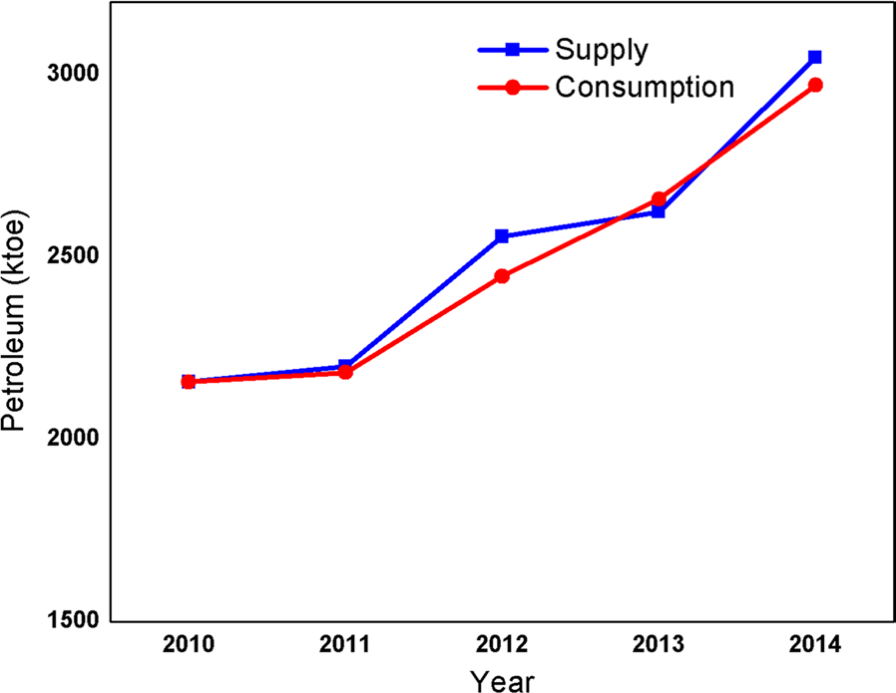
Trend in petroleum supply and consumption from 2010 to 2014

Ethiopia does not have oil deposits and relies entirely on imported petroleum products, either refined or in crude form. The various petroleum products required for end-use purposes mainly in transport, agriculture, commercial and industrial sectors are; liquefied petroleum gas (LPG), kerosene, jet/turbo fuel, petroleum gasoline, diesel, fuel oil, and lubricating oils and greases. The country spends a huge amount of foreign currency to import petroleum products. Petroleum consumption had shown increasing by 1.6% from 2010 (2158 ktoe) to 2014 (2972 ktoe) which is driven by economic growth (see Fig. 6) [12]. Petroleum fuels are mainly used in the transport sector (80% of the total consumption of petroleum products) with a smaller share of the demand from the household sector (kerosene for cooking and lighting) and industrial sector (fuel oil for thermal energy), the total petroleum consumption in 2014 was 2972 ktoe (Fig. 6).

### Renewable energy policy in Ethiopia

Biomass energy's sustainability will depend on the successful management of biomass resources and government policy. Examining the implications of biomass energy use in Ethiopia, it was noted that deliberate policies are required to improve the quality and sustainability of biomass energy in Ethiopia. The need for the policies would be to make clean commercial energy more accessible and relatively cheaper.

Ethiopia has released many policy and strategic documents to ensure that the Sustainable Development Goals (SDGs) are accomplished. The leading ones are the Climate Resilient Green Economy Strategy (CRGE), Ethiopia's National Energy Policy, and the Biomass Energy Strategy. Among these policies and strategies: (a) The Green Economy Strategy has prioritized programs that could help to develop sustainable forestry and reduce demand for fuelwood (i.e., by reducing demand for fuelwood by distributing and using fuel-efficient stoves or by using alternative-fuel cooking and baking techniques such as liquefied petroleum gas (LPG), electric or biogas stoves) that contribute to forest management, enhanced carbon sequestration, reduction of forest degradation, afforestation and reforestation of woodlands [14, 15]. (b) The purpose of the National Energy Policy is to increase sustainable and renewable energy sources (i.e., bioenergy supply) and to increase the efficiency of the use of bioenergy. Its main objective is to improve the efficiency of the use of biomass fuel, promote the move towards greater use of modern fuels, resolve household energy problems by promoting agroforestry, and incorporate environmental sustainability into energy production and supply systems [14]. The policy also states that to increase the availability of electricity, the country will not only rely on hydropower, but will also benefit from other renewable and sustainable energy options, such as solar panels, geothermal energy and wind power. Also, in major energy-consuming sectors, such as transport, industry and others, the country needs to promote energy conservation while ensuring that energy production is environmentally friendly and sustainable and to provide sufficient encouragement to the private sector [16]. (c) The Government of Ethiopia has also developed its sustainable bioenergy policy as an important component of the National Development Program Strategy, with decent legal provisions for the promotion of environmentally friendly energy sources, the distribution and use of biofuels throughout the country, and the replacement of fossil fuels for use in transport sectors and mitigation of climate change.

## Current status of biomass energy potential and utility

**Fig. 7:**
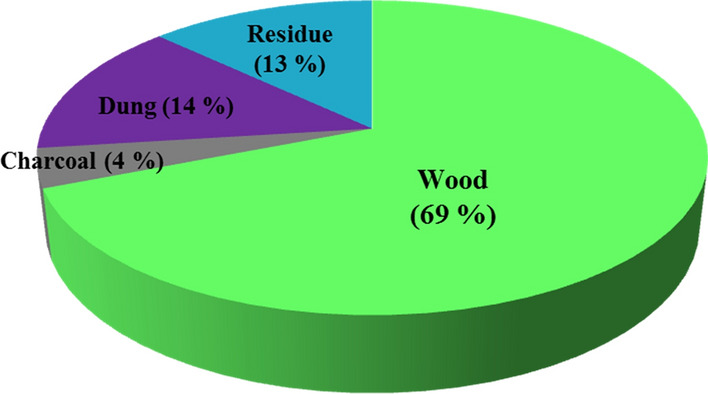
The share of different biomass resources as fuel in Ethiopia, 2013 [20]

Biomass is a natural resource used for various purposes, including energy, all around the globe [17, 18]. In developing countries, especially sub-Saharan countries such as Ethiopia, it is regarded as the backbone of energy sources [19]. Examples of biomass include woody biomass (cellulose, hemicellulose, lignin, lipids, proteins, and simple sugars), residues of crops, animal waste, dung, sewage, agricultural waste, and municipal waste. In Ethiopia, wood, agricultural, animal waste and human waste are the commonly used biomass energy resources. It is estimated that the overall energy that can be produced annually from these resources is around 101,656.77 Tcal. Of this, it is estimated that the share of woody biomass is 73% (wood 69% and charcoal 4%), followed by dung (14%) and residue (13%) (Fig. 7) [20]. The majority of rural society relies on the free collection of woody biomass, residues of crops and animal dung. However, utilization is still being unbalanced, and consumption is greater than re-plantation.

### Different biomass feedstock and their potential for biofuel production

#### Wood and charcoal

Almost all African countries still rely on wood to meet basic energy needs [21–24]. Wood fuels account for 90–98% of the energy consumption in most sub-Saharan Africa [22, 25] Firewood is the cheapest source of energy available that most people use widely [26, 27]. Consisting mostly of fallen sticks or branches, prunings of living or dead branches removed from standing trees, and wood from cut or felled trees, it is sourced from forests, woodlands, shrub lands and in some cases from trees on farms (scattered trees, agroforestry, or energy woodlots. Between 2013 and 2017, the total volume of wood fuel produced globally was about 9.44 billion m^3^ with an average annual production of 1.88 billion m^3^ [22, 28]. Three-quarters of global wood fuel production and consumption is in Africa (35%) and Asia (39%). The tropics and subtropics (i.e., Africa, Latin America, and Asia) hold 88.3% of the global share of wood fuel production. In many developing countries, it is the most dominant source of energy [29]. The percentage of biomass fuels in the total energy consumption in Ethiopia is one of the highest in the world, accounting for over 90% of the total energy consumption in the country and about 99% in the rural areas [21, 30]. It was claimed that the shortage of biomass fuels has been one of the major causes of deforestation and subsequent, land degradation in Ethiopia.

In rural areas, most of the wood demand is fulfilled by collecting, whereas the urban households fulfill most of their wood demand by purchasing. According to the CSA welfare monitoring survey (2011), about 87.2% of the rural households used collected wood and 3.6% purchased wood. Whereas, 18.6% of the urban households consumed collected wood and about 44.7% purchased wood [31]. The standing stock of woody biomass of the country is estimated at 1,150 million tons [31]. Demand for fuelwood is growing rapidly while its supply is shrinking and increasing access distance which leads especially women and children to travel a long distance for collecting it. The principal drivers of wood fuel demand are population growth, lack of access to biomass energy substitutes and the growing rate of poverty among the population. The wood fuel energy supply and demand imbalance is exerting considerable pressure on the remaining forest and vegetation stocks, thereby accelerating the processes of land degradation and deforestation, which is the largest source of GHG emissions in Ethiopia.

In 2010, about 17% of the country’s GHG emission is caused by deforestation for fuelwood [31]. On the other hand, charcoal is an important fuel, particularly for urban dwellers. Its production is however associated with the increasing levels of deforestation. It is a process of carbonization of wood by partial combustion or application of heat from an external source. In Ethiopia charcoal is produced in a very small scale which is about 100 to 300 kg per batch using the earth mound kiln. To produce 1 kg of charcoal about 8 kg of wood is consumed which results in a great deal of waste in this traditional process (i.e., earth mound kiln). Ethiopia has a world share of 8.5% charcoal production and about 47% of the Ethiopian households use charcoal, with 82% of the usage in the urban households, and 34% in the rural households. The total charcoal production in the year 2016 was estimated to be 4.32 million tons [32]. The demand for charcoal has grown faster because of increasing urbanization, increasing monetization of charcoal, and increasing competitiveness of charcoal with kerosene [31].

Household air pollution (HAP) exposure from traditional cooking practices is one of the major killers worldwide among environmental risk factors [33]. Almost 600,000 Africans die annually and millions more suffer from HAP-induced diseases [34]. Improved cook stove (ICS) adoption is key to addressing this public health problem, which mainly affects developing countries where traditional cooking practices are used by many families [35]. In sub-Saharan Africa countries including Ethiopia, adoption of ICS has the potential to generate a variety of health, social, economic, and environmental benefits [10, 36–39]. The adoption of ICS has significantly contributed to improvements in living conditions through wood savings, reduced women's workload by reducing the time required for fuel collection, reducing indoor air pollution, reducing particulate matter (PM) and carbon monoxide (CO), and created self-employment for the stove producers [40]. Among the adopted ICS, Merchaye and Lakech cooker stoves are the popular ones in Ethiopia with differential emissions and fuel use efficiency [40, 41].

#### Agricultural crop residues

Agriculture is the predominant and important economic sector in Ethiopia. The agricultural sector accounts for roughly 43% of GDP, 90% of exports and 80% of total employment in the country. Cereals dominate Ethiopian agriculture, accounting for about 70% of agricultural GDP. Scarcity of wood leads to greater use of agricultural residues and animal dung for cooking which could otherwise have been used to enhance the nutrient status and texture of the soil and contribute positively to agricultural production. Agricultural residues are mostly used by the rural household for cooking and baking, using very low-efficiency cooking stoves. Agricultural residue supply is seasonal and hence its use as fuel is also seasonal. Agricultural residues are seasonal, therefore, collection and storage of residues during the months of availability will be necessary; and alternatively, different residues could be sourced at different times of the year to fill the gap of scarcity [42]. The typical agricultural residues densification process has to undergo several stages including collection, storage, cleaning, drying, and size reduction. Depending on the types of residue, each of the above stages will require a certain expenditure on equipment, materials and labor [42].

#### Animal dung

Animal dung in the form of dung cake is one of the most common traditional biomass used by households for cooking. According to CSA (2009/2010) survey, the country’s livestock population is about 150 million [42]. It is seen that about 42 million tons of dry weight dung is annually produced from the total livestock from which, cattle (cows and oxen) are accounted for the highest share of dung production about 84% of the annual total dung production [42]. Cow dung is the primary source of the substrate for domestic bio-digesters. Over 77% of the rural households in Ethiopia own cattle; hence, they are eligible for bio-digester installation. Rural households lead an integrated crop-livestock agricultural system. Consequently, the integration of the biogas technology with an adopter animal husbandry is central to the adoption process in Ethiopia [43]. In Ethiopia, even if the production of biogas started in the last long year, still there are too much need to optimize the biogas resources, adoption, and technologies that will ease the burden for women and children who spend up to 10 h a week gathering wood in some rural areas to reduce indoor pollution and improve prospects for small farmers [44].

#### Municipal solid waste

**Fig. 8:**
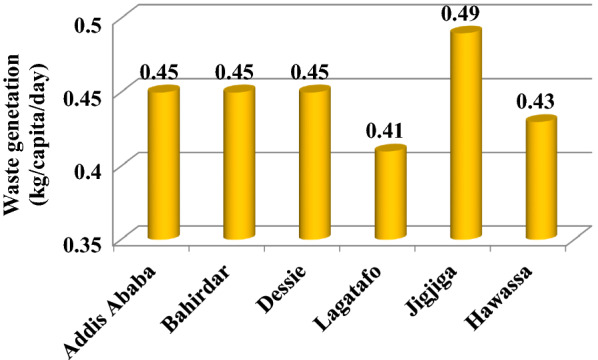
Current waste generation in kg/capita/day of some cities in Ethiopia [45–49]

Municipal waste can be used to produce methane gas, which is then used to generate electricity. The technology for the conversion of waste into electricity is mature and is used in various parts of the world. The amount of municipal solid waste depends on the population of the cities. It is also one of the potential bioenergy resources of Ethiopia accumulated in cities in the form of landfills. The current global generation of waste is approximately 2.01 billion tons per year and is projected to grow to 3.4 billion tons per year by 2050 [45]. It is estimated that total waste generation in Ethiopia is between 0.6 and 1.8 million tons per year in rural areas and between 2.2 and 7 million tons per year in urban areas. The major cities of the country are highly populated; for instance, the population of Addis Ababa was increased from 2.96 million in 2007 to about 6.6 million in 2017 (estimated). With this population increase and economic growth, the municipal solid waste is highly increasing. In Ethiopia, there is an annual rise in waste generation by 5%, according to Ali and Eyasu [46]. The municipal solid waste generation rates for the main cities of Ethiopia are depicted in Fig. 8.

**Fig. 9:**
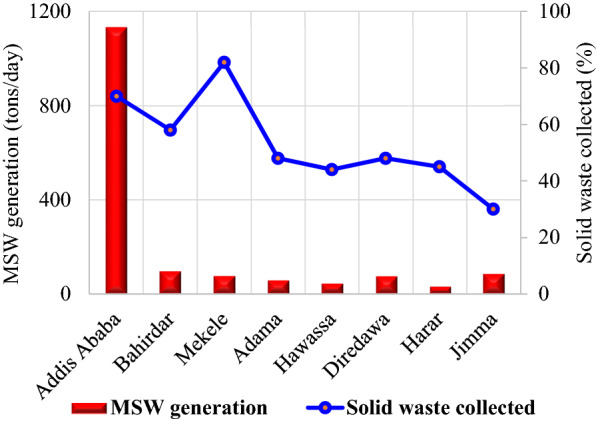
Waste collection in major cities of Ethiopia, 2010 [45, 49]

Considering the daily average municipal solid waste generation rate at 0.45 kg per capita per day (Fig. 8) [46], the daily and annual solid waste output of Addis Ababa would be about 2970 and 1,084,050 tons, respectively, in 2017 (estimated). Municipal solid waste is becoming a threat to the major cities of Ethiopia, as only less than 50% of the waste produced per day was properly collected and disposed of, leaving half of the waste created uncollected or disposed of in unauthorized areas (Fig. 9). In Ethiopia, the efficiency of solid waste management, recycling and disposal systems remains very low [45, 47]. Informal, unregulated, and unhealthy forms are used to recycle a very limited proportion of waste [46]. Waste is frequently burned in open and unregulated ways by households to get rid of the waste. The African Development Bank Group has estimated that more than 50% of the population in Ethiopia is widely involved in the open burning of waste. Recycling is not well-practiced and, because of the absence of formal structure and control, it is at a primitive stage in Ethiopia.

**Fig. 10:**
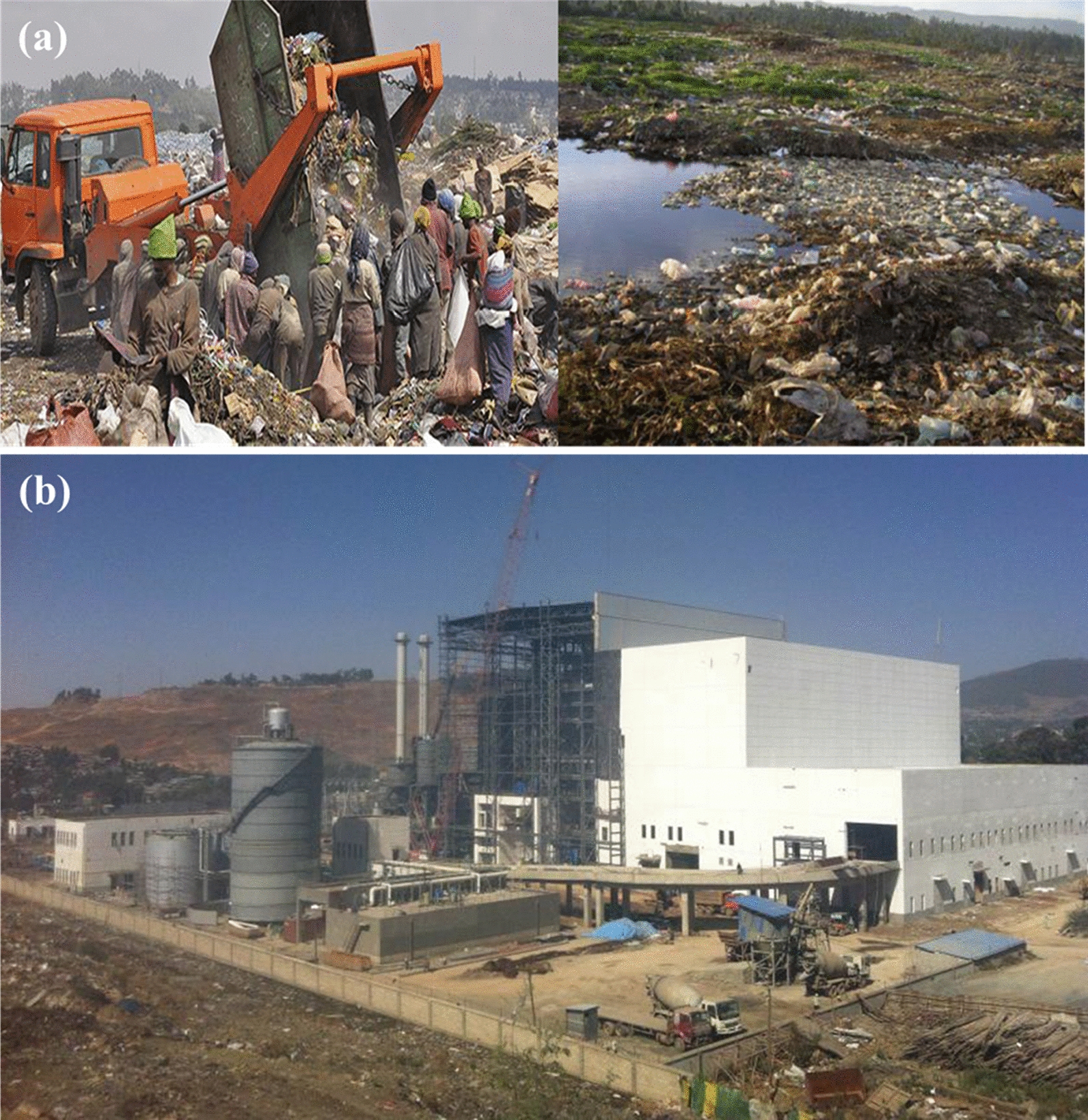
**a** ‘Reppi’, solid waste disposal site and compaction; **b** ‘Reppi’ waste-to-energy power plant

For half a century, the Koshe dump site (37 hectares) has been the only landfill in Addis Ababa (see Fig. 10). In 2017, a landslide on the Koshe dump site killed 114 people, prompting the government to declare three days of mourning. But a new Reppie waste-to-energy (WTE) plant is set to transform the site and revolutionize the entire city’s approach to dealing with waste. WTE describes a variety of technologies that convert garbage or municipal solid waste (MSW) into either heat or electricity. Incineration processes have taken place in the presence of air and at the temperature of 850 °C and waste is converted to carbon dioxide, water and non-combustible materials with solid residue (Bottom ash) [50]. Reppie waste-to-energy is said to be African’s first waste-to-energy facility, which is inaugurated in August 2018, expected to incinerate 1400 tons of waste every day, that’s roughly 80% of the city’s rubbish, all while supplying Addis Ababa with 30% of its household electricity needs and meeting European standards on air emissions (Fig. 10b) [50]*.* The project is the result of a partnership between the Government of Ethiopia and a consortium of international companies: Cambridge Industries Limited (Singapore), China National Electric Engineering and Ramboll, a Danish engineering firm and constructed for US$95 million [50].

#### Biofuel

Biofuel is a fuel derived from biomass. It is an organic matter taken from plants and animals. It comprises mainly wood, agricultural crops and products, aquatic plants, forestry products, wastes and residues, and animal wastes. In its most general meaning, biofuel is all types of solid, gaseous and liquid fuels that can be derived from biomass [51].

Biodiesel and ethanol are the two most commonly used biofuel types. Biodiesel products are potentially trusted substitutes for fossil fuels because they are clean and renewable fuels that can be used without the need to redesign the existing technology in any direct-injection engine [52, 53]. Bioethanol (ethyl alcohol, grain alcohol, CH_3_–CH_2_–OH, or ETOH) is a liquid biofuel that can be produced from various biomass feedstocks and conversion technologies. Bioethanol is an attractive alternative fuel because it is a renewable bio-based resource and it is oxygenated hereby provides the potential to reduce particulate emissions in compression–ignition engines [54]. Bioethanol is a renewable alcohol-based fuel that can be produced from starches, sugars, and cellulosic biomass. Traditional feedstock, which is used for ethanol production, includes crops such as corn, wheat, and sorghum. With recent advances in cellulosic technology; ethanol can also be produced from agricultural waste products like sugar cane bagasse, rice hulls, potato waste, and brewery waste; from forestry and paper wastes; and from municipal solid waste [54]. The raw materials for bioethanol production can broadly be classified as (i) sucrose-containing feedstock (sugarcane, sugar beet, and sweet sorghum), (ii) starch-containing feedstock (wheat, corn, and cassava), and (iii) cellulosic feedstock (straw, grasses, wood, agricultural wastes, paper, etc.) [55]. A summary of the opportunities and challenges of using biofuels is given in Table 2.

**Table 2:** Some advantages and disadvantages of biofuel production and use [56]

Aspects	Advantages	Disadvantages
Cost	It is made from renewable resource	Currently more expensive than fossil diesel fuel
Energy	Fossil diesel fuel is a limited resource, but biofuels can be manufactured	Mainly produced from edible oil, which could cause shortages of food supply and increased prices
Availability	From a wide range of materials	Reduction of fuel economy
GHG emission	Significantly less harmful carbon (CO_2_,CO,TCH) emission	Conflicts with food supply
Energy security	Viability of the first-generation biofuel production relatively less flammable compared to fossil diesel significantly better lubricating properties significantly less harmful carbon emission compared to standard diesel	less suitable for use in low temperature it can only be used in diesel powered engines More likely than fossil diesel to attract moisture
Air pollution	significant reduction of PM emissions	Caused increases in NOx

##### Bioethanol

By investing over 80% of foreign earnings annually, Ethiopia imports its entire petroleum fuel requirement [57]. In general, the transport sector, which accounts for approximately 52% of the country in particular, is one of the most key sectors, consuming the majority of the petroleum imported and contributing more CO_2_ to the environment [57]. Since the economy of the country is growing, demand for petroleum fuel is expected to increase. It is therefore important to look for locally available alternative fuels, such as biofuels, to ensure the country's sustainable development and fuel security. Therefore, the production of biofuels has the potential to meet a significant proportion of national energy needs, minimize reliance on imported fossil fuels, generate new business opportunities and contribute to reducing emissions of greenhouse gases (GHGs). Taking the aforementioned challenges, Ethiopia is currently assessing its biofuel potential and is now in the process of implementing an ambitious biofuel strategy, which was approved in 2007 [58]. Due to the favorable air condition and suitable soil type for biodiesel development, the country grows various types of plant species that can be used for the production of biodiesel. Jatropha, which is a very important biodiesel feedstock, grows in many parts of the country and is also used as a hedge and medicinal plant [58].

In a country like Ethiopia that relies heavily on imported fossil fuels, there are also apparent reasons for promoting biofuels. Biofuels are regarded as an opportunity to ensure domestic energy security, rapid economic growth and wealth creation. There are high expectations that biofuels will contribute to solving the country's main development challenges today [58]. In Ethiopia, almost all the feedstocks needed for the production of bioethanol (sugarcane, sugar-beet, cereals, and maize) are grown. In light of the national policies that discourage the use of food crops as feedstock for food security reasons, the current production of bioethanol is only a byproduct from sugar estates [59]. Ethanol production in Ethiopia is linked with sugar factories and aimed for import substitute of petroleum products, enhance agricultural development and agro-processing, job creation, and export earnings. However, only a small fraction of the potentials are utilized yet and an alternate 5% and 10% ethanol blend has been accessed in the capital city of the country. Moreover, Finchaa and Metehara are the only two sugar factories producing bioethanol in the country [58]. In 2014/15 about 20.5 million liters of ethanol were supplied to the energy system of the country (8 million liters from Fincha sugar factory, whereas about 12.5 million liters per year were from the Metahara sugar factory) and all used in the transport sector [12, 60].

**Fig. 11:**
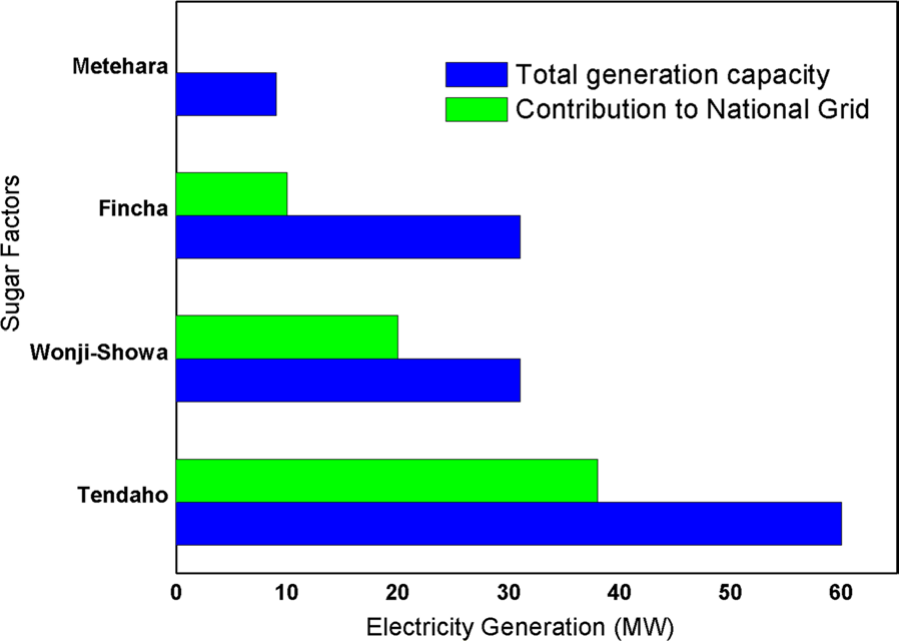
Bagasse energy generation capacity of sugar factories in Ethiopia and their contributions to national grid [60]

Currently, there are three bioethanol blending stations in the country namely, Nile Petroleum, Oil Libya and National Oil Company [42, 61]. On the other hand, bagasse is the byproduct of sugar industries; and from one ton of crushed cane, about 27% to 33% of bagasse can be produced [42, 61]. Bagasse is used for steam production and electricity generation to fulfill the requirement of the mills. Most of the sugar factories contribute bagasse energy for the energy sector of the country, in addition to bioethanol. Among these factories, Tendaho, Wonji/showa, Fincha and Metehara sugar factories produce electricity for their own consumption and contribute to the national grid. These sugar factories have a capacity to produce 60 MW, 31 MW, 31 MW and 9 MW of electric power, respectively. Metehara sugar factories produce 9 MW of electric power and satisfy its own power demand by itself, but Tendaho, Wonji/showa, Fincha sugar factories are contributing to the national grid about 38 MW, 20 MW and 10 MW of electric power, respectively, after they satisfy their own needs (Fig. 11) [60, 61].

##### Biodiesel

Globally, the awareness of energy issues and environmental problems associated with burning fossil fuels has encouraged many researchers to investigate the possibility of using alternative sources of energy instead of oil and its derivatives. Among them, biodiesel seems very promising for several reasons: it is highly biodegradable and has minimal toxicity, it can replace diesel fuel in many different applications such as boilers and internal combustion engines without major modifications, only a small decrease in performances is reported, results in almost zero emissions of sulphates, aromatic compounds and other chemical substances that are destructive to the environment, has only a small net contribution of carbon dioxide (CO_2_) when the whole life cycle is considered (including cultivation, production of oil and conversion to biodiesel), and it appears to cause a significant improvement of rural economic potential [62]. The invention of the vegetable oil fuelled engine by Sir Rudolf Diesel dated back to the 1900s. However, a full exploration of biodiesel only came into light in the 1980s as a result of renewed interest in renewable energy sources for reducing greenhouse gas (GHG) emissions and alleviating the depletion of fossil fuel reserves. Biodiesel is defined as mono-alkyl esters of long-chain fatty acids derived from vegetable oils or animal fats and alcohol with or without a catalyst [63–66]. Compared to diesel fuel, biodiesel produces less sulphur, carbon dioxide, carbon monoxide, particulate matter, smoke and hydrocarbons emission and more oxygen. More free oxygen leads to complete combustion and reduced emission [67]. Biodiesel has been in use in many countries such as the United States of America, Malaysia, Indonesia, Brazil, Germany, France, Italy and other European countries.

Ethiopia is endowed with natural resources suitable for biodiesel development and at the national level, an estimated area of 25 million hectares of suitable land is available for the development of biodiesel [59, 68]. Biodiesel production is necessary for energy security especially in the transport sector which will be achieved by blending biodiesel with diesel so that to decrease consumption of diesel as well as GHG emissions. Electricity generation and cooking fuel are other applications of biodiesel. The byproduct of biodiesel production could also be used to produce soaps and cosmetic products [59].

#### Biogas

Biogas is a combustible mixture of gas. It consists mainly of methane and carbon dioxide and is made from biodegradation of organic material under anaerobic conditions. It is a methane-rich fuel gas produced by anaerobic digestion of organic materials with the help of methanogenic bacteria. Some of the biogas-producing materials (substrates) range from animal dung to household, agricultural and industrial wastes [69]. Biogas technology offers a very attractive route to utilize certain categories of biomass for meeting partial energy needs [70–73]. It provides an alternative energy source to the use of traditional fuel sources, which is dominantly used in most developing countries. Biogas technology serves two major purposes, biogas and bio-slurry. Biogas energy could replace the use of firewood, charcoal and kerosene for cooking, heating and lighting while bio-slurry could replace the use of chemical fertilizer for agricultural production [74]. However, key informants and user households viewed that the cooking and bio-fertilization perspectives of the technology have been overlooked due to the unavailability of efficient biogas cooking stoves for baking and inadequate training for bio-slurry management. Findings from previous studies show that the African continent utilizes very little of the potential of biogas technology due to the inability to exploit its full potential [75, 76].

An ambitious goal to install two million domestic bio-digesters by 2020 is set by the African Biogas Initiative [77]. With the support of this initiative, in Rwanda, Tanzania, Kenya, Uganda, Ethiopia, Cameroon, Benin and Burkina Faso, national biogas programs in Africa have been implemented [78]. By the end of 2009, nearly 300,000 fixed-dome bio-digesters with volumes ranging from 4 m^3^to 15 m^3^had been built in Africa [61]. The National Biogas Programme of Ethiopia (NBPE) is part of the SSA's implementation of biogas technology that is gaining momentum due to the African Biogas Initiative [79]. The NBPE was implemented with the participation of various development partners, such as the Ministry of Foreign Affairs of the Netherlands, SNV, GIZ (German Technical Cooperation), HIVOS, the Winrock International Institute for Agricultural Development (US NGO) and the Biogas Institute of the Ministry of Agriculture of China [61, 70, 76, 80–82].

**Fig. 12:**
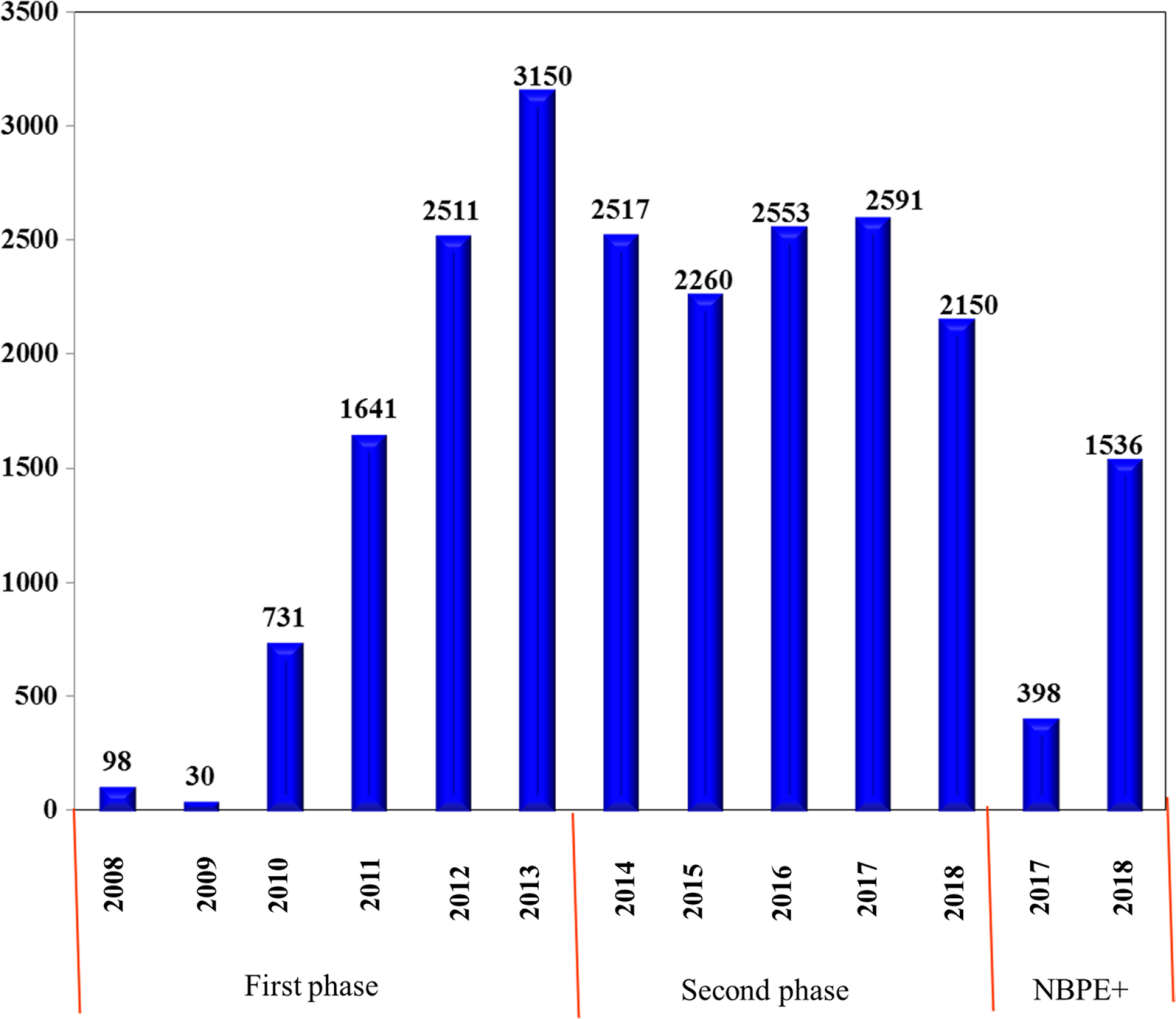
Yearly distribution of biogas digesters in Ethiopia [70, 80, 81, 83]

It was launched in 2008 and planned to install over 30,000 bio-digesters in two phases. The first phase was implemented between 2008 and 2012 and the second phase was between 2013 and 2017. It was planned to develop 14,000 family-sized biogas digesters in the first phase, but only 8161 biogas digesters were built during this phase, including 2480 bio-digesters in Oromia, 1992 in Tigray, 1892 in Amhara and 1699 in SNNPRR [70, 80, 81, 83]. During this phase, only 58% of the planned targets were achieved. Factors such as economic uncertainty, cement crisis, poverty and illiteracy, among others, influenced the dissemination of the first phase. The goal of the second phase of the NBPE was to construct 20,000 additional biogas digesters. In this phase, a total of 12,071 biogas digesters were built [80] (Fig. 12).

Only about 70% of the planned target was accomplished in two phases, with the second phase improving significantly. The slight improvement in the second stage may be attributed to lessons learned from the first phase. The reasons for the failure to achieve the planned goals and the low adoption rate were identified by key informants as technical, financial and institutional challenges [70, 80]. According to Sime (2020), these challenges include the limited technical skill of installation and maintenance service masons, weak institutional responsibilities of implementation units, insufficient and high maintenance service, poor and malfunctioning success stories, and the unwillingness of users to own and maintain installed digesters. In addition, the major obstacles constraining the implementation of the NBPE are high initial investment costs, inflation in the cost of raw materials for the construction and installation of bio-digesters, and limitations in the size of loans [70, 80, 84–86]. Similarly, in SSA, inadequate distribution strategies, lack of project monitoring and follow-up by promoters, poor ownership responsibility by users, are major challenges to biogas technology domestication programs [87]. Cost consequences, lack of coordination and the negative image of the technology caused by past failures are important challenges for biogas technology programs [80, 88]. Meanwhile, key informants stated that the Government of Ethiopia aims to develop a private biogas sector that is autonomous, sustainable and market-oriented. A National Biogas Dissemination Scale-up Program (NBPE+) is currently being introduced by the NBPE, which will continue to 2022 and covers all regional states of the country [80].

### Different technologies of biomass conversion to bioenergy production

There are various conversion technologies available, from biomass to electricity. Thermochemical conversion, biochemical conversion and physicochemical conversion have been generally categorized [89–93]. This section reviews the advancement of Biomass Conversion Technologies in Ethiopia.

#### Thermochemical conversion of biomass

Energy is created by the application of heat and chemicals in the processes of thermochemical conversion. The four current thermochemical conversion processes are combustion, pyrolysis, gasification, and liquefaction [89].

##### Combustion

**Fig. 13:**
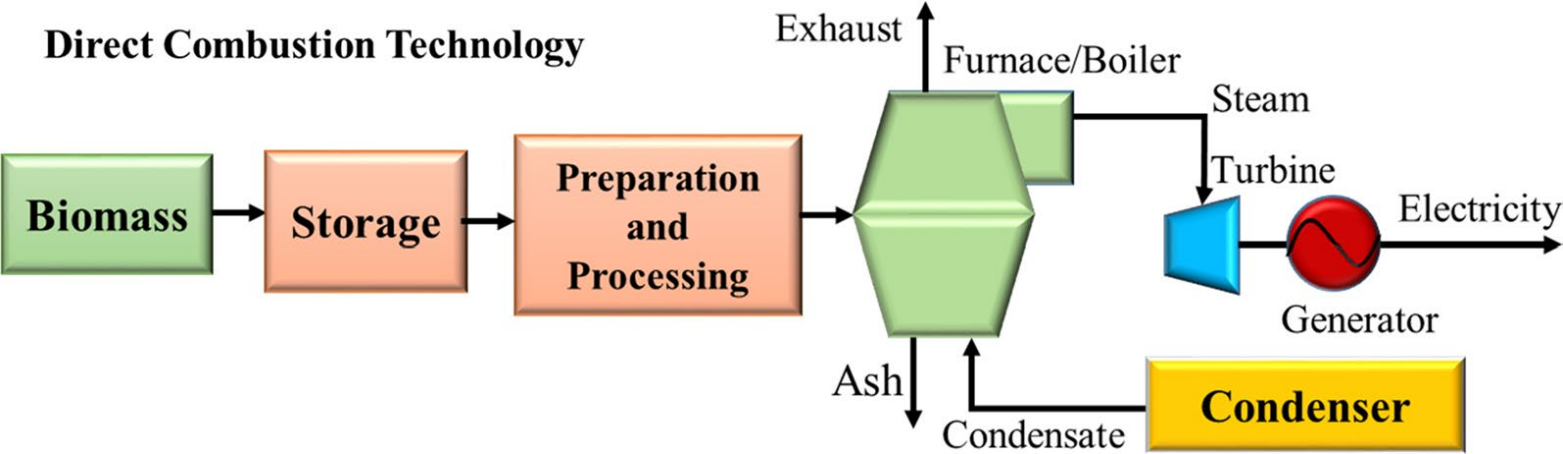
Biomass combustion scheme

This conversion technology generates approximately 90% of the total biomass capacity. In this method, biomass is burned at high temperatures in a combustion or furnace to produce hot gas, which is then fed into a steam producing boiler, which is expanded to generate mechanical or electrical energy via a steam turbine or steam engine (Fig. 13). The technology is capable of operating on various biomass types, i.e., wood, dry leaves, hard vegetable shells, rice husk, dried animal dung, etc. The combustion process is an exothermic chemical reaction, i.e., the biomass is burned in the presence of air with the resulting release of chemical energy that could be transformed into mechanical and electrical energy [89, 94, 95].

The majority of biomass energy generation in Ethiopia is obtained through combustion processes, but the efficiency of these processes is very low, resulting in energy waste. In the country, biomass combustion is primarily used in rural poor communities to provide energy for cooking. This method is characterized by slow, inefficient three-stone stoves with high specific fuel consumption. Other processes include the use of charcoal stoves.

##### Pyrolysis

**Fig. 14:**
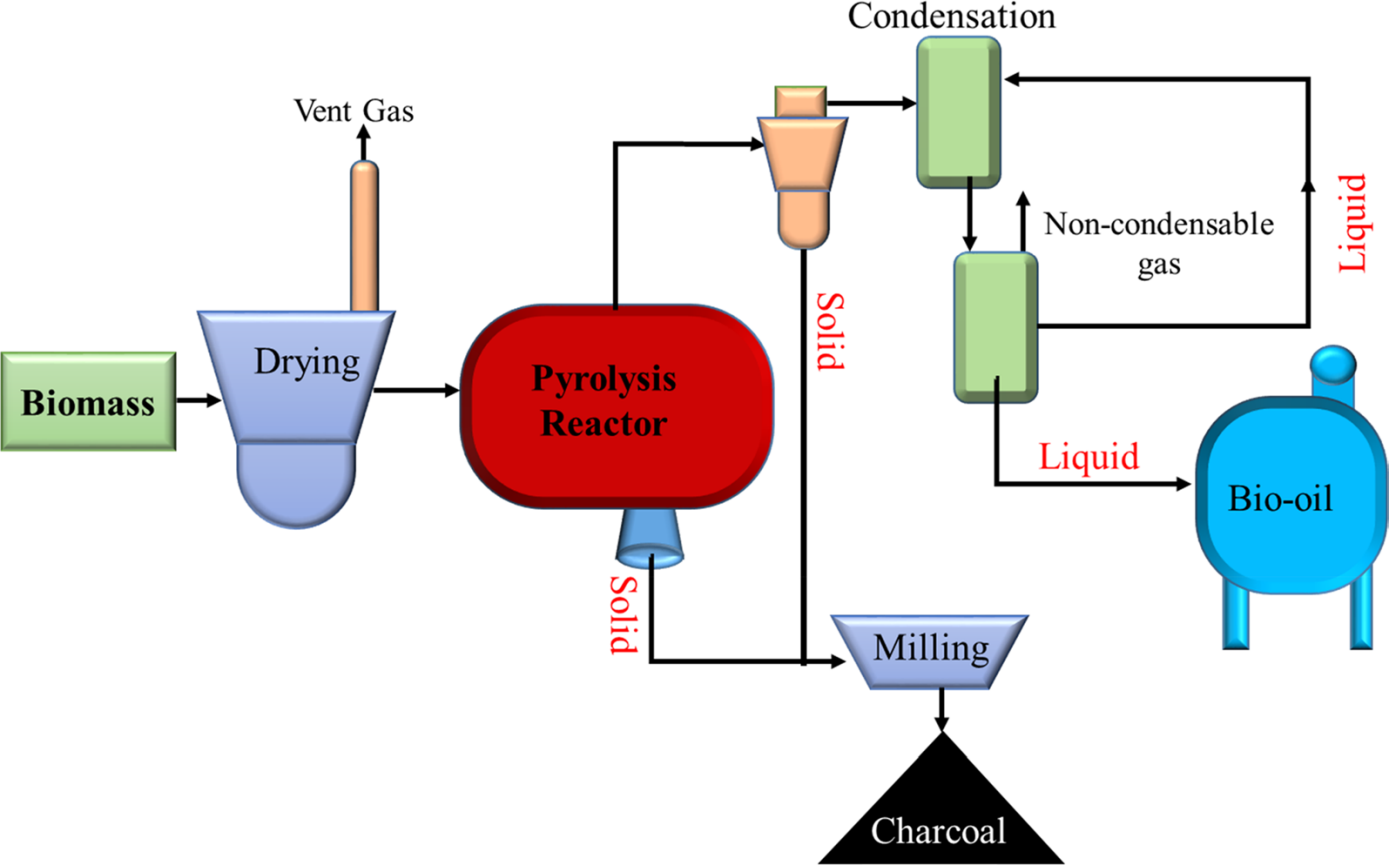
Biomass pyrolysis scheme

Pyrolysis is the heating of biomass at temperatures within the 500 °C–900 °C range in the absence of oxygen in a closed vessel [94]. It produces liquid (bio-oil), solid (charcoal), and gaseous (combustible gas). High temperatures cause the volatile components of the biomass producing gases to be vaporized, the vapors of which are condensed by liquefaction into liquids (Fig. 14). The liquid fuel resulting from this process can be stored and subsequently used for different applications for heating and generating electricity [89, 95]. Biomass pyrolysis has only been limited in Ethiopia at the research and development level and plant evaluation. There were also a few feasibility studies on the potential for cogeneration from wood residues and agricultural residues.

##### Gasification

**Fig. 15:**
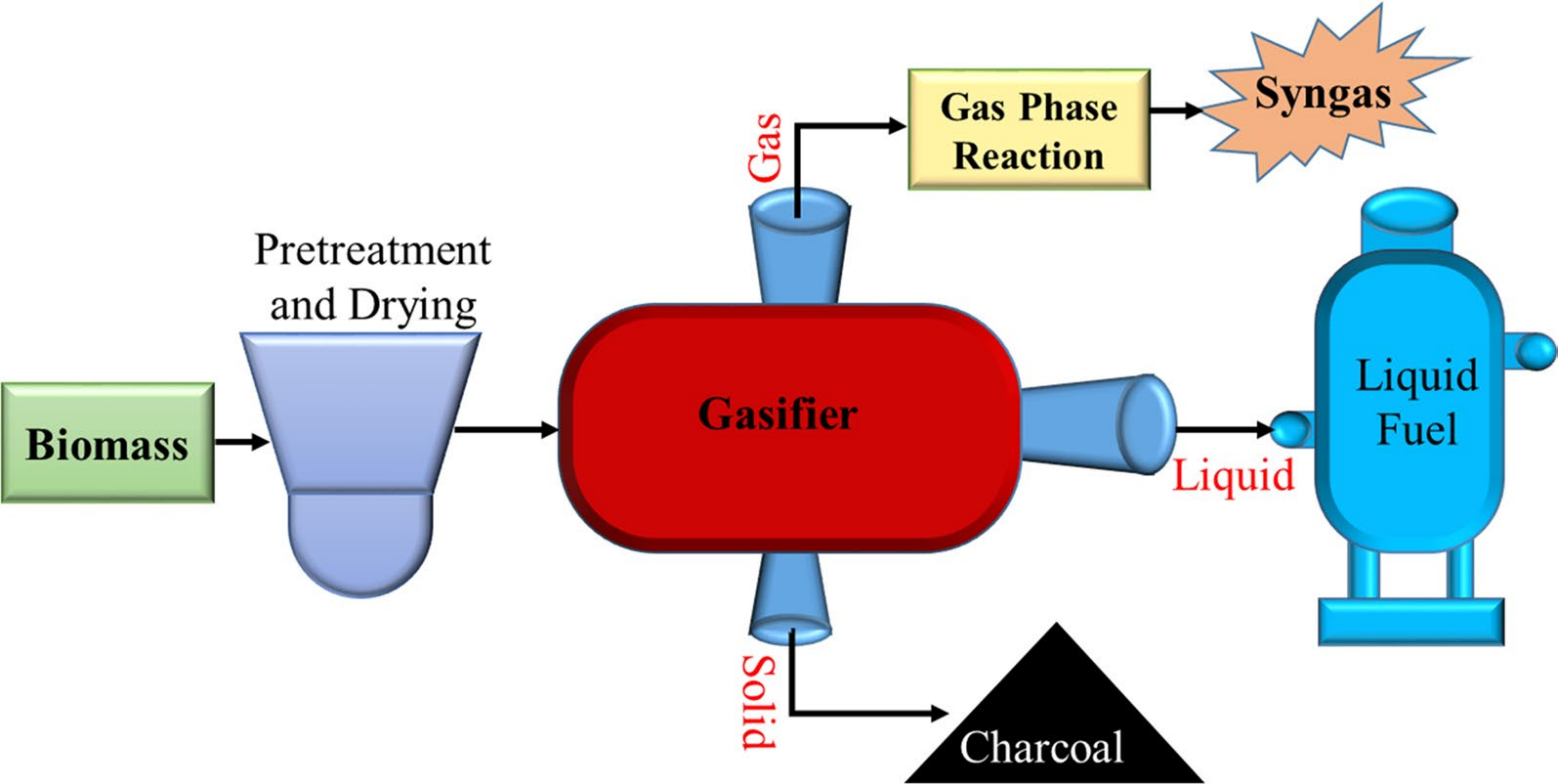
Biomass gasification scheme

The gasification process is carried out by heating solid biomass with minimal oxygen/air (O_2_ and air deficient) to produce gas of low heating value or by reacting with steam and oxygen to produce medium heating value, called synthesis gas or syngas, mainly composed of CO, hydrogen (H_2_), CH_4_ and nitrogen (N_2_), at high pressure and temperature. Syngas can be used as an electricity-generating fuel or as a source for a large range of petrochemical and refining products, such as methanol, ammonia, synthetic gasoline (Fig. 15), etc. [96]. Like pyrolysis, biomass gasification in Ethiopia has also been limited only at the R&D and plant evaluation level, and a few feasibility studies have also been conducted on the potential for cogeneration from wood residues.

##### Liquefaction

**Fig. 16:**
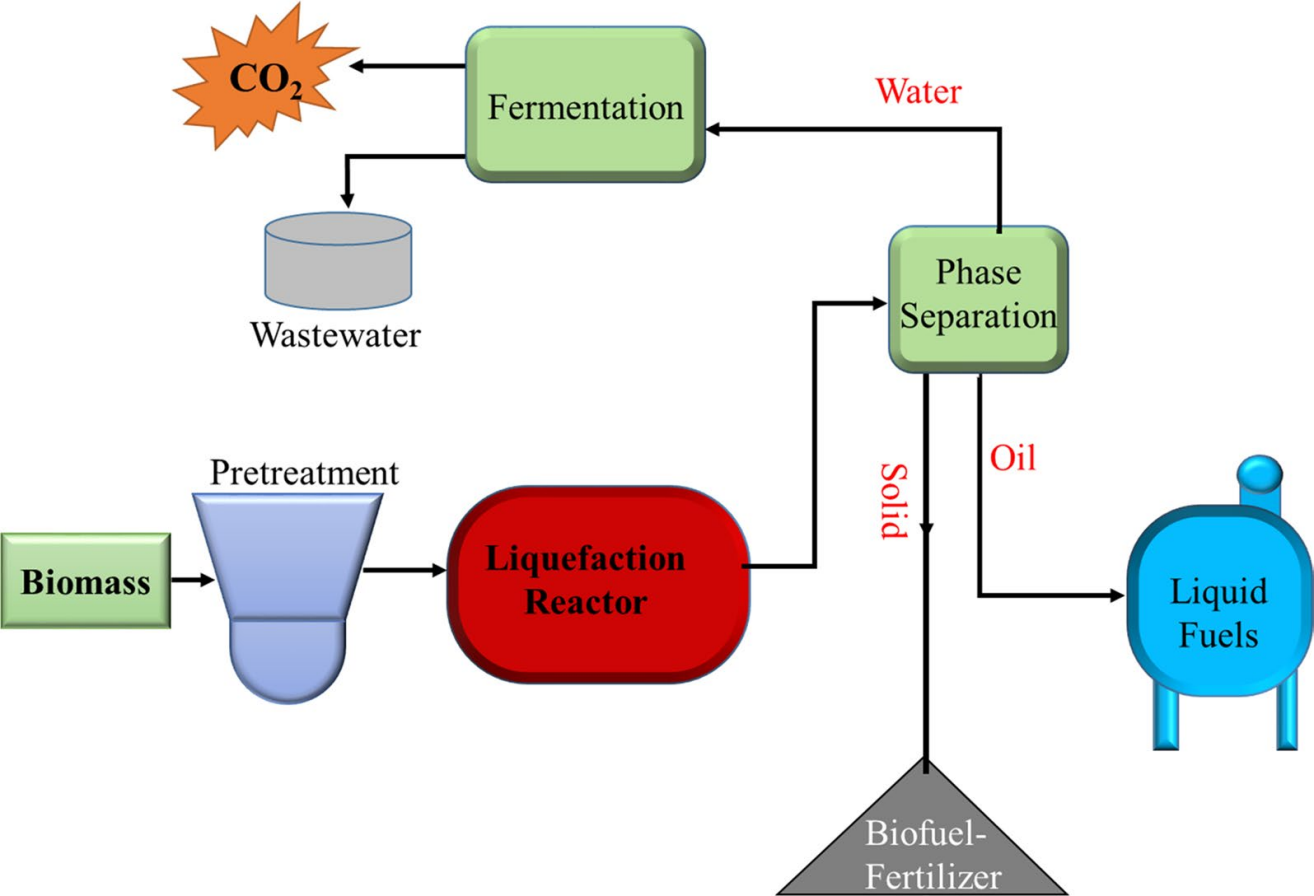
Biomass liquefaction scheme

Liquefaction is a method of biomass conversion performed at moderate temperatures between 280 and 370 °C and high pressures (10–25 MPa in water). Liquid bio-granulates, similar to crude oil, are also produced, as are other gaseous, aqueous and solid by-products (Fig. 16). The products obtained have a high heating content and low oxygen content, making it a chemically stable fuel. The main purpose of liquefaction is to produce oil that has a high H/C ratio [89, 97–99]. This biomass conversion technology in Ethiopia is still in its infant stage and is still under research and development as the other technologies.

#### Biochemical conversion of biomass

To break down biomass, biochemical conversion processes use enzymes from bacteria and other microorganisms. Biochemical conversion processes for biomass include anaerobic digestion and fermentation.

##### Anaerobic digestion

**Fig. 17:**
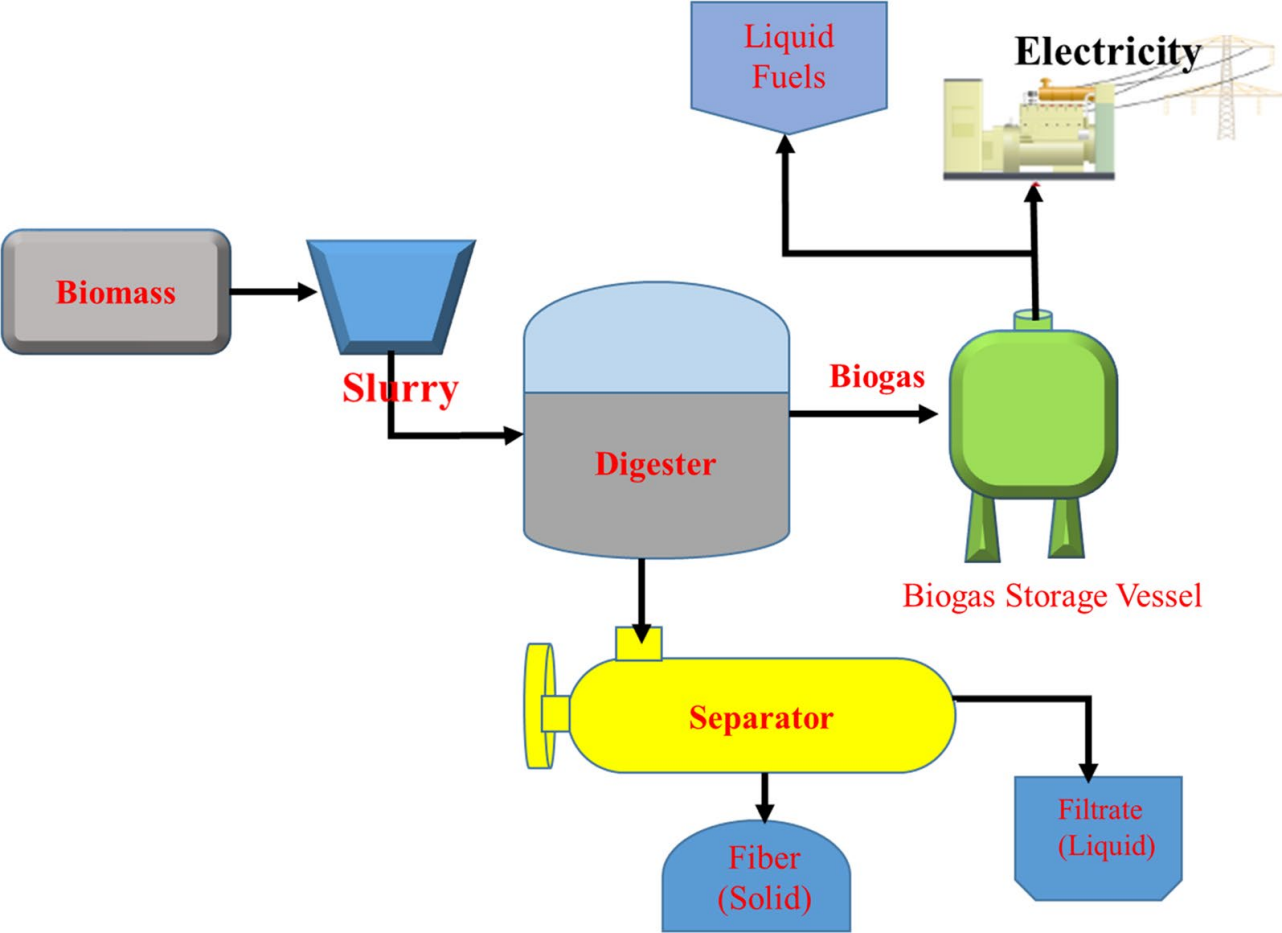
Biomass anaerobic digestion scheme

In the absence of oxygen, anaerobic digestion creates biogas from wet organic substrates. Hydrolysis, acidogenesis, acetogenesis, and methanogenesis are the four basic stages of this process. Throughout the process, microorganisms in an oxygen-free environment enable a series of chemical reactions to take place via natural metabolic pathways (Fig. 17) [89, 100]. Sewage sludge, agricultural residues, MSW, and animal manure are some of the feedstocks commonly used in this type of process. To utilize a biogas technology in Ethiopia some scientific, engineering, and economic-based research works have been carried out at the institutional level. The NBPE was introduced in 2008 and over 18,000 bio-digesters were able to be installed in two stages. The NBPE has designated a diverse group of actors within this evolving biogas sector to contribute to the implementation of biogas technology [61, 70, 76, 80–82].

##### Fermentation

**Fig. 18:**
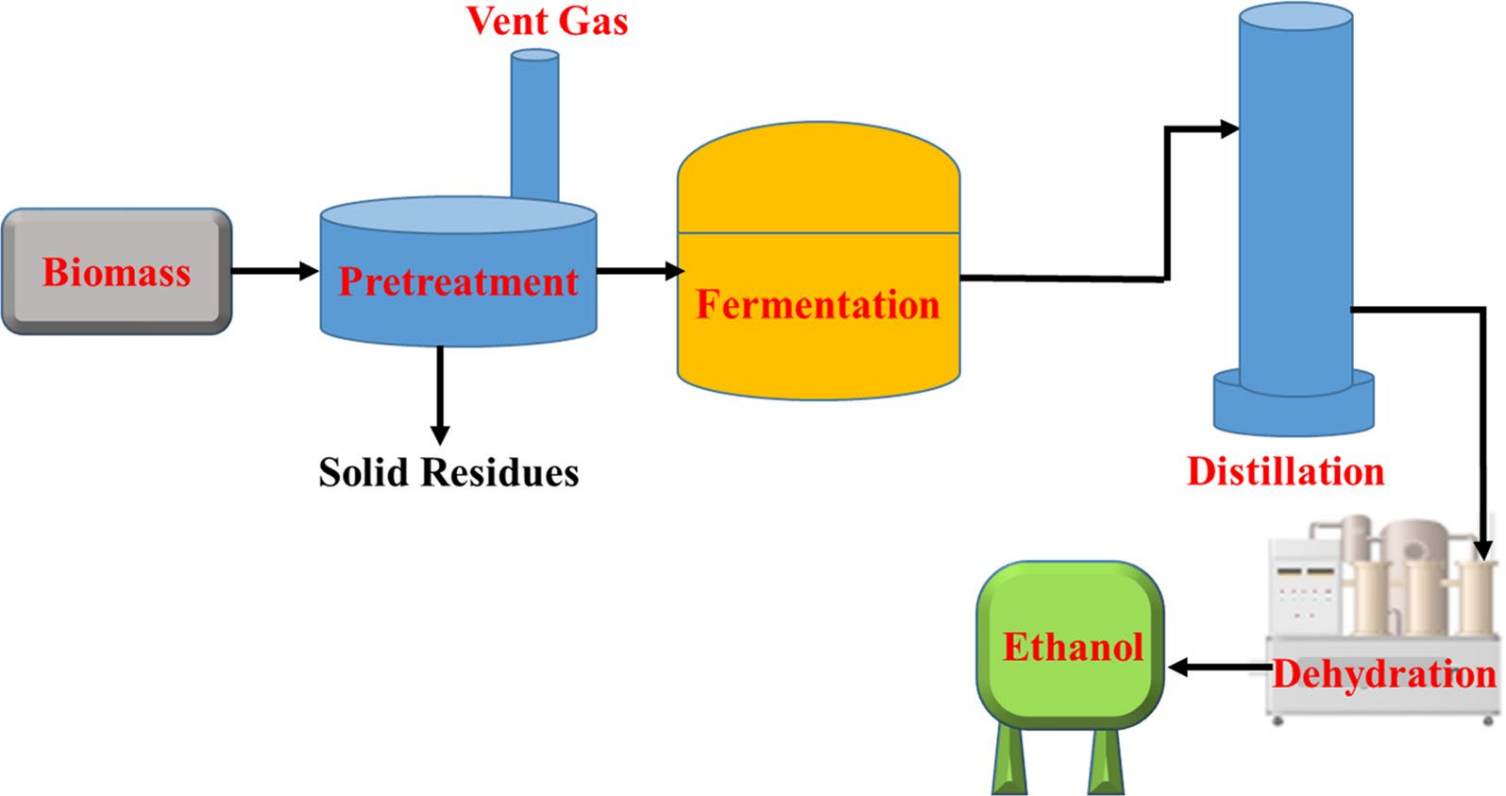
Biomass fermentation scheme

Fermentation is the mechanism where a number of microorganisms transform carbohydrates, such as starch and sugar, into ethanol (Fig. 18). The biomass is ground down and the starch is converted to sugars by enzymes, with yeast then converting the sugars to ethanol. *Saccharomyces cerevisiae* are the most common microorganisms used in the process, and the feedstock used for this type of process is divided into three categories: sugars, starch, and lignocellulosic substrates. Distillation is an energy-intensive step that produces approximately 450 L of ethanol from 1000 kg of dry corn. The solid residue from this process can be given to cattle, and the bagasse from sugarcane can be used for subsequent gasification or as a fuel for boilers [90, 100]. About 8 million liters of bioethanol is produced annually in Ethiopia using molasses as feedstock. The country also aims to blend 5% ethanol into its gasoline pipeline. The feasibility of using ethanol for domestic purposes such as cooking and heating is being investigated by a UNDP project in the region.

#### Physicochemical conversion of biomass

**Fig. 19:**
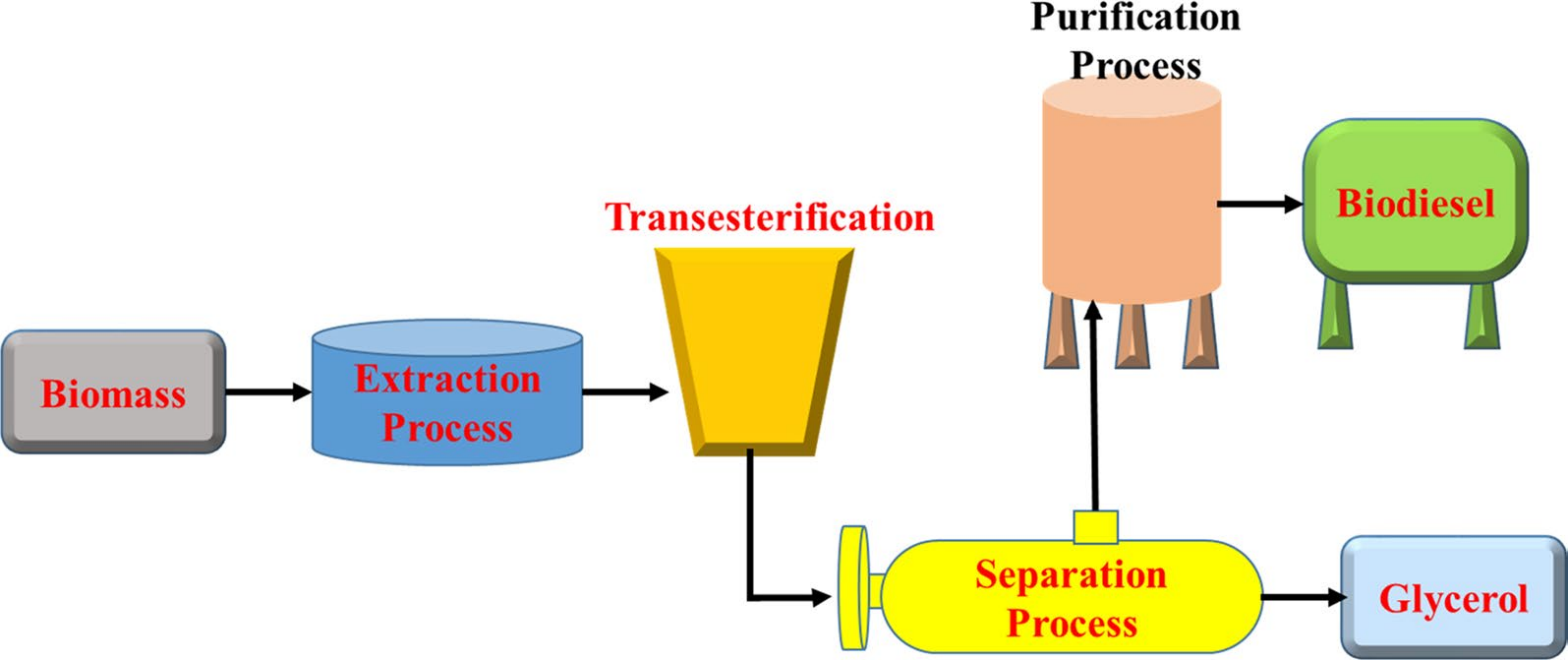
Biomass physicochemical conversion scheme

Biomass processes of physicochemical conversion lead to the production of high-density biofuels (Fig. 19). More specifically, through esterification and/or transesterification processes, different forms of vegetable oil and animal fats are converted to biodiesel. Rapeseed oil and sunflower oil constitute 80–85% and 10–15% of total biodiesel production worldwide, respectively, are major vegetable oils used to manufacture first-generation biodiesel [89]. For the production of second and third generation biodiesel, waste oils, including waste cooking oil (WCO) and microbial oil, including algal oil, may also be used. It's worth noting that oils are mostly composed of triglycerides, which aren't usable fuels. In fact, the transformation of crude vegetable oil is required because otherwise, problems such as incomplete combustion and subsequent residue accumulation in engines are likely. As a result, the raw material must be processed further, primarily through transesterification, in order to separate the triglyceride molecules into their constituents, fatty acids and glycerol. The triglycerides are converted into methyl or ethyl esters (biodiesel) by using methyl or ethyl alcohol (in excess) in the presence of mostly an alkaline catalyst during the transesterification reaction [100, 101]. The production and use of liquid biofuels as alternative fuels to fossil fuel is a recent phenomenon in Ethiopia. Generally, the main interest has been in biodiesel derived from *Jatropha curcas*, palm oil, and castor bean. Some initiatives on biofuel development have already been taken by the government, the private sector, non-governmental organizations (NGOs), and the UNDP.

### Life-cycle analysis, economic perspective, and bio-refinery approach

The potential of biomass to produce high-value-added products has sparked the interest of various research groups involved in biofuels, food and feed, and pharmaceuticals [102, 103]. These characteristics make biomass feedstocks, particularly microalgae, a viable candidate for bio-refinery exploitation. However, before further research into potential industrialization is conducted, a comprehensive life-cycle analysis (LCA) is required. LCA quantifies all of the resources required for biomasses planting/cultivation, harvesting, extraction, and purification, as well as the emissions and environmental impact of the same process. Furthermore, an economic analysis of the entire bio-refinery approach is required to understand the viability of biomass as a feedstock. These tools help to understand current scenarios and generate different paths to commercial industrialization of biomass bio-refineries. LCA is evaluated using two indicators: global warming potential (GWP) and net energy ratio (NER). The amount of CO_2_ emitted per unit of energy is used to calculate the GWP. Ideally, all greenhouse gases would be considered for this quantification, but the literature data are limited to CO_2_ emissions. NER is calculated using the process's total energy flow. It is the ratio between the energy required to obtain the final products from biomass and the total energy stored in the final product [102].

Aside from the Life Cycle Assessment, the economic feasibility of biomass-based bio-refineries is also critical for commercialization. For example, Hoffman et al. [104] conducted a cost–benefit analysis of biodiesel production using Algal Turf Scrubber (ATS) and Open Raceway Ponds (ORP). Their findings revealed that the cost of producing biodiesel from ATS and ORP is $8.34 and $6.27 per gallon of biodiesel, respectively, despite the fact that these prices do not provide positive economic feasibility. Dasan et al. [105] used three different cultivation systems to obtain biodiesel and other by-products from a different fraction of microalgae feedstock (open pond/raceway pond, bubble column PBR, and tubular PBR). The capital cost of tubular and bubble column PBRs is higher than the operation cost, accounting for nearly 47.5–86.2% of the total cost, according to an economic feasibility analysis based on the production of 100,000 kg of biomass for 340 days of the year. However, operation and maintenance account for 45.73% of the total cost in an open ponds cultivation system. The production of bioethanol as a byproduct was examined in this study, but the complex and expensive processes involved in bioethanol production do not favor economic profitability. In contrast, Lam et al. [106] predicted that the highest total revenue generated from microalgae biomass is around €31 per kg of dry weight, compared to a production cost of €6–7 per kg of dry weight. However, these figures can only be achieved if the cost of downstream processing is kept to a minimum. Developing simpler and cost-effective downstream processing techniques appears to be critical for achieving the economic feasibility of biomass bio-refinery systems.

## Opportunities, challenges and prospects of biomass energy

### Opportunities

Biomass for waste water treatment: among the challenging environmental problems owing to their toxic effects and possible accumulation throughout the food chain and hence in the human body are pollution leaked by organic and inorganic contaminants. Besides, many hazardous compounds (metals, dyes, phenolic compounds, etc.) have found widespread use in industries such as metal finishing, leather tanning, electroplating, nuclear power, textile, pesticide and pharmaceutical. Thus, water pollution by these contaminants is of considerable concern around the world [107–112]. Conventional methods (bioaccumulation, precipitation, reverse osmosis, oxidation/reduction, filtration, evaporation, ion exchange and membrane separation) used for the removal of hazardous compounds from wastewater are expensive and/or inefficient in reducing the effluent concentration to the required levels. The search for new and low-cost techniques is therefore of great importance for the removal of organic and inorganic contaminants from drinking water and wastewater [107–112].

Biosorption, which represents a biotechnological innovation as well as a cost-effective and excellent tool for sequestering hazardous compounds from aqueous solutions, is becoming a potential alternative to traditional treatment processes used for the removal of hazardous metals and organic compounds. It is a term that describes the property of some biomolecules or types of biomass to remove and concentrate by passive binding, selected metallic ions or other molecules from aqueous solutions [107–112]. This implies that the removal mechanism is not metabolically controlled. Biomass exhibits this property, acting just like a chemical substance, for example, an ion exchanger of biological origin. The cell wall structure of certain algae, woody biomass, mosses, fungi and bacteria, in particular, are found to be responsible for this phenomenon [107, 112–114]. In addition, bacteria, fungi, seaweeds, agricultural waste and raw plants can also produce biomolecules having coagulating/flocculating activities. Indeed, the use of biological materials for the treatment of wastewaters containing organic and inorganic contaminants is growing. This relatively new technology has received considerable attention in recent years as it has many advantages over traditional methods. It uses inexpensive and abundant renewable materials with good ability for the recovery of metal pollutants. Thus, studies on the use of biomass such as agricultural wastes, mosses, fungi, bacteria or seaweeds, as a raw material for the production of sorbents is progressively increasing.

Biosorbents including algae, fungi, bacteria and yeasts are investigated for their ability to sequester contaminants; algal biomass has proven to be highly effective as well as reliable and predictable in the removal of hazardous compounds from aqueous solutions [107]. Marine algae are renewable natural biomass and are very abundant in the coastal world. Using as new supports to concentrate and adsorb hazardous compounds, these biomasses have attracted the attention of many investigators as organisms to be tested.

Biological waste gas treatment: there are strong arguments for the development and use of new and original processes to control waste gas emissions from agricultural, industrial, or domestic activities to protect human health and welfare, and also the environment at large. Thus, international treaties for environmental protection (Rio, Kyoto) have been transcribed and applied in many countries. For instance, local legislation particularly for solid waste management, water and wastewater treatment, and air quality has been written based on these ratifications of international agreements. Air pollution control regulations reflect the concern of governments for the protection of people and the environment. The two fundamental reasons for cleaning up the waste gas stream are profit and protection. This is practically when upgrading of biogas, cleaning of waste incinerator flue gas [115], or treating of industrial process emissions.

To remove non-particulate pollutants from a gas stream different processes involving different mechanisms [116, 117] could be achieved based on the nature of the contaminants and/or the complex mixture of pollutants in the gaseous phase, for example, their concentrations and the flow to be cleaned. These processes can be classified into three categories: (1) thermal and/or catalytic oxidation, biological transformation; (2) transfer into a liquid phase (absorption) or onto a solid phase (adsorption) with or without chemical reactions such as acid–base interaction, oxidation, complexation, physisorption or chemisorption and (3) phase change (condensation).

One of these technologies will be chosen with the aim of achieving the required performance for the lowest investment and operating costs depending on the emission characteristics in terms of concentrations and flow. These processes are widely used in industrial applications to remove single toxins or a mixture of contaminants. Many activities including chemistry, petrochemistry, pharmacy, cosmetics, surface cleaning, polymer production, printing, painting, mechanical and car manufacture, and waste and wastewater treatments are concerned.

Biological treatments of gas streams are relatively recent technologies compared with thermal destruction or mass transfer systems. However, researchers have been paying attention to these promising and interesting processes for several years and indeed bioprocesses appear to be a very competitive way to treat the waste gas stream before its discharge into the atmosphere. The removal of a large number of soluble and biodegradable volatile organic compounds (VOCs) or odorous molecules has been the subject of many previous studies and industrial applications [118] 119. The optimal range of pollutant concentration goes from a much diluted pollutant present in the gas stream (from some mg m^−3^ to mg m^−3^) to above 1 g m^−3^. The installation designs cater for an airflow from a few m^3^ h^−1^ to 100,000 m^3^ h^−1^, or even more in some systems.

Hydrogen production from biomass derivatives over heterogeneous photocatalysts: hydrogen storage energy is among the recent development of environmentally benign, renewable and sustainable energy production for the near future. Hydrogen is a storable energy carrier with a high energy content and non-polluting nature, which can be effectively converted into electricity by a fuel cell or into motive power by a hydrogen-fueled engine without any emission other than water. Even though hydrogen is an attractive alternative energy source, about 96% of the hydrogen supplied currently is derived from fossil fuels such as natural gas (49%), crude oil (29%) and coal (18%) using thermal chemical processes and gasification at high temperature [120]. Hydrogen produced from fossil fuels cannot be regarded as really an environmentally benign fuel because it takes a very long time to regenerate fossil fuels and the consumption of fossil fuel increases the concentration of carbon dioxide in the atmosphere contributing to global warming. For the realization of a sustainable society, hydrogen needs to be produced from renewable resources and natural energy like biomass energy.

Biomass (e.g., plants, starch and oil) and its derivatives (e.g., ethanol, glycerol, sugars and methane) have attracted much attention as the best candidate for hydrogen sources among the renewable resources. If the biomass and its derivatives are consumed for hydrogen production with carbon dioxide formation, the produced carbon dioxide from biomass and its derivatives can be converted again into biomass through plant photosynthesis. This means that the carbon dioxide produced from the biomass should not, in principle, contribute to global warming (i.e., it is carbon–neutral) when the consumption of the biomass does not exceed the natural capacity for conversion of carbon dioxide to biomass.

Thermal gasification and biological hydrogen production by fermentation are the two major approaches extensively studied as methods to convert biomass into hydrogen. Although these are promising hydrogen production methods, there are major problems to be solved for practical appreciation. For instance, thermal gasification requires high reaction temperatures at 1073–1273 K and thus consuming considerable amounts of energy while the reaction rate of biological hydrogen production is quite low that results in low productivity. Photocatalytic hydrogen production from water and biomass derivatives is another possible hydrogen production method from biomass [121, 122]. This system is very attractive since hydrogen can be produced at room temperature using sunlight and a photocatalyst. Research on photocatalytic hydrogen production from biomass began in the early 1980s. Since then various attempts have been made to achieve efficient hydrogen evolution.

### Challenges

Some challenges need to be considered in the effort to use biomass energy in Ethiopia, which includes:

Lack of comprehensive national biomass policy and regulation: there is a lack of well-thought and comprehensive policies that direct activities in the biomass energy sector. When there is a requirement to promote the growth of particular renewable energy technologies, policies might be declared that do not adhere to the plans for the development of renewable energy. There is no defined framework for the biomass sector [31].

Weak Institutional coordination: There is an absence of competent institutions with strong mandates and long-term oriented action plans. Institutes, agencies stakeholders who work under the development of biomass energy show poor inter-institutional coordination. Progress in the production of biomass energy is limited by this lack of collaboration, coordination, and delays. Owing to weak coordination, the delay in implementing policies has limited investors' interest in investing in this field. There are some shortcomings in the pre-feasibility reports prepared by the concerned states, which could affect small developers, i.e., local developers, who are willing to undertake projects in the field of biomass energy, in particular biogas. For the creation of renewable infrastructure, proper or well-established research centers are not available and also customer service centers are not available to guide developers concerning renewable projects [31].

Air pollution: a major cause of air quality deterioration and health risks is the smoke that is created from the burning of wood fires. Many women who use firewood as cooking fuel are exposed to smoke, posing a health risk that can lead to respiratory diseases.

Food insecurity: the crops used as energy crops, such as sugar cane, corn, maize, etc., are primarily food crops. Using them for energy production, therefore, results in competition with food production, especially at a time when there is a need to grow more food to feed the population and bring down rising food prices [51].

Forest degradation: the country's rapidly increasing population creates increased demand for firewood and charcoal from a decreasing supply that results in the degradation of forest hectares and other vegetation types [123].

Inadequate transfer of technology and localization: the majority of energy technology hardware is imported due to insufficient technology transfer and underdeveloped manufacturing industries, leading to high foreign exchange spending. There is, for example, a lack of equipment and infrastructure for the storage of biogas for cooking purposes and its conversion into electricity for the population's use, particularly in rural areas [31].

Land availability and right: the bioenergy industry requires large land for the energy corps to plant. Current communal land ownership, with pockets of private ownership, would be an obstacle to large-scale cultivation, which could impact the supply of raw materials for the production of bioenergy [31].

### Prospect of using biomass

Despite these problems, Ethiopia has prospects for the use of biomass resources, including:

Integrative policy and strategy: even if the country has a bioenergy development unit, but so far the formulated national bioenergy policy and strategy are not available. Therefore, it should be formulated and responsible for this unit. Agricultural, forestry, water, food protection, environmental, rural development, financial and other aspects that are important to bioenergy production should be incorporated into the bioenergy policy and strategy. Policies will be more successful if they are specifically related to the target and should be competitively directed towards technological change and the use of biomass. In the long run, policies should reduce greenhouse gas emissions, promote rural development and decrease poverty. In order to encourage bioenergy access, policy and strategies should contribute to the decentralization and devolution of powers to the locals. In developing the bioenergy sector, the government should collaborate with civil society, the private sector and the international community. To ensure market development for bioenergy, it is necessary to promote public–private partnerships and incentives-based bioenergy policies. It is also very important to establish action plans, followed by implementation and monitoring and evaluation.

In addition to the direct effects of bioenergy development, bioenergy policy should deal with indirect environmental and social effects. The formulation of the bioenergy policy is a cross-cutting topic and should include policies on agriculture, forestry, the atmosphere and land use. Adequate consultation and assessment of the environmental impacts of the value chain of the bioenergy type must be carried out. It should be a broad participatory process involving all stakeholders. The policy should be broad-based and promote and encourage the production of bioenergy, education and training, research and development, transport and infrastructure, as well as incentives for producers, distributors and consumers.

Dissemination of information, institutional coordination and stakeholder engagement: Government should disseminate to farmers, investors and lending agencies, planning authorities, forest owners and local communities information and tools for implementing bioenergy projects. Such information and tools may include business models, models of ownership and financing. Such data and resources may include business models, models of ownership and financing. Priorities should be given to institutional coordination and inclusive stakeholder participation. Ministries such as the Ministry of Water, Irrigation and Energy, the Ministry of Agriculture, the Ministry of Finance, the Ministry of Education and the Ministry of Science and Technology should participate in all matters relating to the development of bioenergy in the country. It is important to engage and consult stakeholders, such as chiefs and their local communities, local municipal authorities, civil societies, farmers and forestry associations, local and foreign investors with an interest in bioenergy.

Bioenergy and feedstock value chains: a comprehensive analysis on bioenergy value chains, the availability of feedstock for bioenergy production and food security needs to be performed. It is important to decide exactly how much can be tapped from each bioenergy form and from which feedstock and in which area. The need for foliage, animal feedstock and bioenergy feedstock be assessed and compared. It is important to accurately determine the competing needs for food, bioenergy production and other needs. It is also worth evaluating the relevant technology and its prices.

Research and development: to identify environmental and social risks such as soil erosion, loss of biodiversity, water resource stress, tradeoffs in food supply and impacts of land use change, the government should conduct research through different stakeholders like, universities, scientific and industrial research institutes, agricultural, livestock, and soil. It is very important to communicate and report research outcomes to stakeholders and the general public. To assess direct and indirect effects, complete life-cycle analyses should be performed. The government should not hurry to develop bioenergy, but first, take the appropriate steps to assess the risks involved in the bioenergy sector growth. Prioritize the mitigation of climate change, enhancement of energy security and research and development. This would make it possible to have a sustainable bioenergy sector. Some of the research-requiring areas include but are not limited to, land-use reform, feedstock capital, feedstock transformation technologies, financial schemes and marketing frameworks, mandates and blending targets, and an integrated holistic national strategy with clear bioenergy roles.

## Conclusions

Biomass energy has been the oldest kind of energy utilized by humans as a source of fuel for many years. It is considered as renewable energy source because, unlike carbon-emitting fossil fuels, it is a carbon–neutral energy source. This is why there are breakthroughs and advancements in biomass energy, particularly in the current usage of biomass as a source of energy in many countries. It is an important source of energy, providing more than 80% of Ethiopia's energy consumption. Forest residues, agricultural crop residues, livestock manure, and municipal solid wastes are Ethiopia's primary biomass resources. Electricity access is limited in Ethiopia because the majority of the population lives in rural areas, owing to the country's dispersed population distribution, despite the fact that Ethiopia has a large potential for various alternative energy sources. Furthermore, because national grids were located far from the residents of rural communities, the majority of rural communities lacked daily access to electricity. The majority of rural societies rely on the free collection of woody biomass, crop residues, and livestock dung. As a result, they rely on traditional biomass energy sources for cooking, heating, and lighting, such as burning wood, dung, and agricultural waste. Currently, the demand for energy is increasing, while the supply of power generation must be balanced with the demand. Therefore, this review describes the current dependence on traditional biomass energy types, its impact, and the biomass resources currently available in Ethiopia, as well as their potential for use in the production of various biofuel types. This would help to solve the gap between demand and supply of energy and encourage sustainable delivery of renewable energy to rural communities. Moreover, wastewater treatment, biological waste gas treatment, and hydrogen generation from biomass derivatives over heterogeneous photocatalysts are highlighted among the various prospects for utilizing biomass energy at a big scale and developing biomass energy. We sincerely hope that our contributions to this review will be of great value to researchers, instructors, decision-makers, practicing professionals, senior undergraduate and graduate students, and others who are interested in pollution remediation and energy production and storage using renewable and low-cost bio resources.

## Data Availability

Not applicable.

## Abbreviations

ALRI: Acute lower respiratory infections
COPD: Chronic obstructive pulmonary disease
CRGE: Climate Resilient Green Economy Strategy
GHG: Greenhouse gases
HAP: Household air pollution
ICS: Inter-connected system
LDCs: Least developed countries
LPG: Liquefied petroleum gas
NBPE: National Biogas Programme of Ethiopia
SCS: Self-contained system
SDGs: Sustainable Development Goals
SSA: Sub-Saharan African

## References

[1] Chaturvedi V, Hejazi M, Edmonds J (2013). Climate mitigation policy implications for global irrigation water demand. Mitig Adapt Strateg Glob Chang.

[2] Adams S, Klobodu EKM, Apio A (2018). Renewable and non-renewable energy, regime type and economic growth. Renew Energy.

[3] Bahadori AD-SM (2021). Energy, architecture, and sustainability.

[4] Wu Y, Zhao F, Liu S (2018). Bioenergy production and environmental impacts. Geosci Lett.

[5] Dingeto Hailu A, Kalbessa Kumsa D (2021). Ethiopia renewable energy potentials and current state. AIMS Energy.

[6] International Energy Agency. Africa Energy Outlook: A focus on the energy prospects in sub-Saharan Africa. Paris Cedex 15, France. 2014.

[7] Goldemberg J, Teixeira Coelho S (2004). Renewable energy - Traditional biomass vs. modern biomass. Energy Policy.

[8] IRENA. Renewable Energy Statistics 2016. The International Renewable Energy Agency, Abu Dhabi, UAE. 2016.

[9] Berhanu M, Jabasingh SA, Kifile Z (2017). Expanding sustenance in Ethiopia based on renewable energy resources – A comprehensive review. Renew Sustain Energy Rev.

[10] IEA. Energy Access Outlook2017 From Poverty to Prosperity: World Energy Outlook Special Report. 2017.

[11] Perera A. Electricity in Ethiopia: EEG Energy Insight. UK; 2018.

[12] M. of water and energy of F. D. republic of Ethiopia. Energy Balance and statistical for years 2008/9–2013/14. Addis Ababa, Ethiopia. Addis Ababa, Ethiopia; 2015.

[13] Mondal AH, Bryan E, Ringler C (2018). Ethiopian energy status and demand scenarios: prospects to improve energy efficiency and mitigate GHG emissions. Energy.

[14] Susanne Geissler, Dietmar Hagauer (PM), Alexander Horst, Michael Krause PS. Biomass Energy Strategy Ethiopia; 2013.

[15] Howell J. Chapter 6 Rural Electrification & Renewable Energy in Ethiopia. Environ Policy Rev. 2011;2011: 1–29

[16] Energypedia Ethiopia Energy Situation. https://energypedia.info/wiki/Ethiopia_Energy_Situation#Overview. Accessed 1 Aug 2016.

[17] Long H, Li X, Wang H, Jia J (2013). Biomass resources and their bioenergy potential estimation: a review. Renew Sustain Energy Rev.

[18] Perea-Moreno M-A, Esther Samerón-Manzano A-JP-M (2019). Biomass as renewable energy: worldwide research trends. Sustain Artic.

[19] Kaygusuz K (2012). Energy for sustainable development: a case of developing countries. Renew Sustain Energy Rev.

[20] Gabisa EW, Gheewala SH (2018). Biomass and Bioenergy Potential of bio-energy production in Ethiopia based on available biomass residues. Biomass Bioenerg.

[21] Damte A, Koch SF, Mekonnen A (2012). Coping with fuelwood scarcity. household responses in rural Ethiopia. Environ Dev Disucssion Pap Ser.

[22] Iiyama M, Neufeldt H, Dobie P (2014). The potential of agroforestry in the provision of sustainable woodfuel in sub-Saharan Africa. Curr Opin Environ Sustain.

[23] Bembridge TJ, Tarlton JE (1990). Woodfuel in ciskei: a headload study. South African For J.

[24] Ullah W, Tareen K, Dilbar MT (2020). Present status and potential of biomass energy in pakistan based on existing and future renewable resources. Sustain Rev.

[25] Klas Sander, Besnik Hyseni Waqar WH. Wood-Based Biomass Energy Development for Sub-Saharan Africa. Washington, D.C., U.S.A; 2011.

[26] Lauri P, Havlík P, Kindermann G, et al. Woody biomass energy potential in 2050. Energy Policy; 2013. p. 1–13.

[27] Van HS, Brown M, Srivastava SK (2020). A review on the potential of forest biomass for bioenergy in Australia. Energies.

[28] Tucho GT, Nonhebel S (2017). Alternative energy supply system to a rural village in Ethiopia. Energy Sustain Soc.

[29] Chamberlin J, Jayne TS, Headey D (2014). Scarcity amidst abundance? Reassessing the potential for cropland expansion in Africa. Food Policy.

[30] Sola P, Cerutti PO, Zhou W (2017). The environmental, socioeconomic, and health impacts of woodfuel value chains in Sub-Saharan Africa: A systematic map. Environ Evid.

[31] Beyene GE, Kumie A, Edwards R, Troncoso K. Opportunities for transition to clean household energy in Ethiopia: Application of the WHO Household Energy Assessment Rapid Tool (HEART). World Health Organization; 2018.

[32] Alem S, Duraisamy J, Legesse E (2010). Wood charcoal supply to Addis Ababa city and its effect on the environment. Energy Environ.

[33] Smith KR, Bruce N, Balakrishnan K (2014). Millions dead : how do we know and what does it mean ? methods used in the comparative risk assessment of household air pollution. Annu Rev Public Health.

[34] Kammila S, Kappen JF, Rysankova D, Hyseni B PV. Clean and Improved Cooking in Sub-Saharan Africa. A Landscape Report. Washington (DC). 2014.

[35] Kumar P, Mehta S (2016). Poverty, gender, and empowerment in sustained adoption of cleaner cooking systems : Making the case for refined measurement. Energy Res Soc Sci.

[36] Gebreegziabher Z, Van KGC, Van SDP (2017). Technological innovation and dispersion : Environmental bene fi ts and the adoption of improved biomass cookstoves in Tigrai, northern Ethiopia. Energy Econ.

[37] Gizachew B, Tolera M (2018). Energy for Sustainable Development Adoption and kitchen performance test of improved cook stove in the. Energy Sustain Dev.

[38] Abadi N, Gebrehiwot K, Techane A, Nerea H (2017). Links between biogas technology adoption and health status of households in rural Tigray, Northern Ethiopia. Energy Policy.

[39] Legesse W, Derese A, Samuel T (2015). Determinants of Adoption of Improved Stove Technology in Dendi district, West Shoa, Oromia Regional State, Ethiopia. Am J Hum Ecol.

[40] Mamuye F, Lemma B, Woldeamanuel T (2018). Emissions and fuel use performance of two improved stoves and determinants of their adoption in Dodola, southeastern Ethiopia. Sustain Environ Res.

[41] Damte A, Koch SF. Clean Fuel-Saving Technology Adoption in Urban Ethiopia Clean Fuel-Saving Technology Adoption in Urban; 2011.

[42] Mengistu AT. Modeling and Analysis of Long-Term Shifts in Bioenergy Use (With Special Reference to Ethiopia). Royal Institute of Technology, KTH. MSc Industrial Engineering; 2013.

[43] Eshete G, Sonder K, Heegde F. Report on the feasibility study of a national programme for domestic biogas in Ethiopia. Netherland; 2006.

[44] Mengistu MG, Simane B, Eshete G, Workneh TS (2016). Factors affecting households’ decisions in biogas technology adoption, the case of Ofla and Mecha Districts, northern Ethiopia. Renew Energy.

[45] Teshome FB. Municipal solid waste management in Ethiopia ; the gaps and ways for improvement. J Mater Cycles Waste Manag. 2020.

[46] Mohammed A, Elias E (2017). solid waste management environmental impacts in Addis Ababa city. J Environ Waste Manag.

[47] Regassa N, Sundaraa RD, Seboka BB (2018). Challenges and opportunities in municipal solid waste management: the case of Addis Ababa City, Central Ethiopia. J Hum Ecol ISSN.

[48] Fenta BA (2017). Waste management in the case of Bahir Dar City near Lake Tana shore in Northwestern Ethiopia: a review. African J Environ Sci Technol.

[49] Getahun T, Mengistie E, Haddis A (2012). Municipal solid waste generation in growing urban areas in Africa : current practices and relation to socioeconomic factors in Jimma, Ethiopia. Env Monit Assess.

[50] Abebe MA (2018). Challenges of Waste to Energy Facility in Reppi (koshe), Addis Ababa City. Int Res J Pharm Med Sci.

[51] Negash M, Swinnen JFM (2013). Biofuels and food security : Micro-evidence from Ethiopia. Energy Policy.

[52] Anwar M (2021). Biodiesel feedstocks selection strategies based on economic, technical, and sustainable aspects. Fuel.

[53] Athar M, Zaidi S (2020). A review of the feedstocks, catalysts, and intensification techniques for sustainable biodiesel production. J Environ Chem Eng.

[54] Muktham R, Bhargava SK, Bankupalli S, Ball AS (2016). A review on 1st and 2nd generation bioethanol production-recent progress. J Sustain Bioenergy Syst.

[55] Balata M, Havva Balata CO (2008). Progress in bioethanol processing. Prog Energy Combust Sci.

[56] Datta A, Hossain A, Roy S (2019). An overview on biofuels and their advantages and disadvantages. Asian J Chem.

[57] Hiben YG. Long-term Bioethanol Shift and Transport Fuel Substitution in Ethiopia Yacob Gebreyohannes Hiben Transport Fuel Substitution in Ethiopia. Master of Science Thesis KTH School of Industrial Engineering and Management Energy Technology EGI-2013-ECS; 2013.

[58] Gebreegziabher Z, Mekonnen A, Ferede T (2014). Profitability of Biofuels Production The Case of Ethiopia. Environ Dev Discuss Pap Ser.

[59] Gebreegziabher Z, Mekonnen A, Ferede T, Kohlin G (2017). Profitability of Bioethanol Production: the Case of Ethiopia. Ethiop J Econ.

[60] Ethiopian Sugar Corporation (ESCo). New Sugar Development Projects. http://www.etsugar.gov.et/en/projects/item/23-new-sugar-development-projects.html. Accessed 20 Jun 2004

[61] Williamson L, N’Goran K, Labriet M. Sustainability of biogas and solid biomass value chains in ethiopia: Results and recommendations from implementation of the Global Bioenergy Partnership Indicators. Addis Ababa, Ethiopia. 2019.

[62] Ahmad AL, Yasin NHM, Derek CJC, Lim JK (2011). Microalgae as a sustainable energy source for biodiesel production: a review. Renew Sustain Energy Rev.

[63] Janaun J, Ellis N (2010). Perspectives on biodiesel as a sustainable fuel. Renew Sustain Energy Rev.

[64] Satyanarayana M, Muraleedharan C (2011). A comparative study of vegetable oil methyl esters (biodiesels). Energy.

[65] Shahid EM, Jamal Y (2011). Production of biodiesel: a technical review. Renew Sustain Energy Rev.

[66] Kafuku G, Mbarawa M (2010). Biodiesel production from Croton megalocarpus oil and its process optimization. Fuel.

[67] Fazal MA, Haseeb ASMA, Masjuki HH (2011). Biodiesel feasibility study: an evaluation of material compatibility; performance; emission and engine durability. Renew Sustain Energy Rev.

[68] Taddese H (2014). Suitability analysis for Jatropha curcas production in Ethiopia - a spatial modeling approach. Environ Syst Res.

[69] Akinbami JFK, Ilori MO, Oyebisi TO (2001). Biogas energy use in Nigeria: current status, future prospects and policy implications. Renew Sustain Energy Rev.

[70] Manon L, Bermúdez E (2016). Ethiopia’s emerging domestic biogas sector : Current status, bottlenecks and drivers. Renew Sustain Energy Rev.

[71] Rittmann BE (2008). Opportunities for renewable bioenergy using microorganisms. Biotechnol Bioeng.

[72] Faisal S, Hafeez FY, Zafar Y (2018). A review on nanoparticles as boon for biogas producers-Nano fuels and biosensing monitoring. Appl Sci.

[73] Chala B, Oechsner H, Latif S, Müller J (2018). Biogas potential of coffee processing waste in Ethiopia. Sustainability.

[74] Luostarinen S, Luste S, Sillanpää M (2009). Increased biogas production at wastewater treatment plants through co-digestion of sewage sludge with grease trap sludge from a meat processing plant. Bioresour Technol.

[75] Rupf GV, Bahri PA, De Boer K, McHenry MP (2015). Barriers and opportunities of biogas dissemination in Sub-Saharan Africa and lessons learned from Rwanda, Tanzania, China, India, and Nepal. Renew Sustain Energy Rev.

[76] Shallo L, Ayele M, Sime G (2020). Determinants of biogas technology adoption in southern Ethiopia. Energy Sustain Soc.

[77] Clemens H, Bailis R, Nyambane A, Ndung’u V,  (2018). Africa Biogas Partnership Program: A review of clean cooking implementation through market development in East Africa. Energy Sustain Dev.

[78] Morris GA. Bioenergy for sustainable development in Africa. In: Bioenergy for sustainable development in Africa. Springer Science+Business Media, South Africa, 2012. p 1–413.

[79] Ghimire PC (2013). SNV supported domestic biogas programmes in Asia and Africa. Renew Energy.

[80] Sime G, Tilahun G, Kebede M (2020). Assessment of biomass energy use pattern and biogas technology domestication programme in Ethiopia. African J Sci Technol Innov Dev.

[81] Gabisa EW, Gheewala SH (2019). Biomass and Bioenergy Potential, environmental, and socio-economic assessment of biogas production in Ethiopia : The case of Amhara regional state. Biomass Bioenerg.

[82] Mengistu MG, Simane B, Eshete G, Workneh TS (2015). A review on biogas technology and its contributions to sustainable rural livelihood in Ethiopia. Renew Sustain Energy Rev.

[83] Gezahegn TW, Gebregiorgis G, Gebrehiwet T (2018). Adoption of renewable energy technologies in rural Tigray. Ethiopia : An analysis of the impact of cooperatives.

[84] Mengistu MG, Simane B, Eshete G, Workneh TS (2016). The environmental benefits of domestic biogas technology in rural Ethiopia. Biomass Bioenerg.

[85] Berhe M, Hoag D, Tesfay G, Keske C (2017). Factors influencing the adoption of biogas digesters in rural Ethiopia. Energy Sustain Soc.

[86] Kelebe HE (2018). Returns, setbacks, and future prospects of bio-energy promotion in northern Ethiopia: The case of family-sized biogas energy. Energy Sustain Soc.

[87] Edem Cudjoe Bensah AB-H (2010). Biogas technology dissemination in Ghana: history, current status, future prospects, and policy significance. Int J Energy Environ.

[88] Roopnarain A, Adeleke R (2017). Current status, hurdles and future prospects of biogas digestion technology in Africa. Renew Sustain Energy Rev.

[89] Mckendry P (2002). Energy production from biomass ( part 2): conversion technologies. Bioresour Technol.

[90] Kumar A, Kumar N, Baredar P, Shukla A (2015). A review on biomass energy resources, potential, conversion and policy in India. Renew Sustain Energy Rev.

[91] Mboumboue E, Njomo D (2018). Biomass and Bioenergy Biomass resources assessment and bioenergy generation for a clean and sustainable development in Cameroon. Biomass Bioenerg.

[92] Prasad S, Radhakrishnan S, Kumar S, Kannojia S. Chapter 9 Sustainable Energy : Challenges and Perspectives. In: Sustainable Green Technologies for Environmental Management. Springer Nature, Singapore, 2019. p 175–197.

[93] Toklu E (2017). Biomass energy potential and utilization in Turkey. Renew Energy.

[94] Lebaka V (2013). Potential bioresources as future sources of biofuels production: An Overview.

[95] Kaushika ND, Reddy KS, Kaushik K (2016). Biomass Energy and Power Systems. Sustainable Energy and the Environment: A Clean Technology Approach.

[96] Rahimpour MR, Arab Aboosadi Z, Jahanmiri AH (2012). Synthesis gas production in a novel hydrogen and oxygen perm-selective membranes tri-reformer for methanol production. J Nat Gas Sci Eng.

[97] Gollakota ARK, Kishore N, Gu S (2018). A review on hydrothermal liquefaction of biomass. Renew Sustain Energy Rev.

[98] Rowbotham J, Dyer P, Greenwell H, Theodorou M (2012). Thermochemical processing of macroalgae: a late bloomer in the development of third-generation biofuels?. Biofuels.

[99] Yu G, Zhang Y, Schideman L (2011). Distributions of carbon and nitrogen in the products from hydrothermal liquefaction of low-lipid microalgae. Energy Environ Sci.

[100] Tursi A (2019). A review on biomass: Importance, chemistry, classification, and conversion. Biofuel Res J.

[101] Leung DYC, Wu X, Leung MKH (2010). A review on biodiesel production using catalyzed transesterification. Appl Energy.

[102] Koyande AK, Show PL, Guo R (2019). Bio-processing of algal bio-refinery: a review on current advances and future perspectives. Bioengineered.

[103] Faried M, Samer M, Abdelsalam E (2017). Biodiesel production from microalgae: Processes, technologies and recent advancements. Renew Sustain Energy Rev.

[104] Hoffman J, Pate RC, Drennen T, Quinn JC (2017). Techno-economic assessment of open microalgae production systems. Algal Res.

[105] Dasan YK, Lam MK, Yusup S (2019). Life cycle evaluation of microalgae biofuels production: Effect of cultivation system on energy, carbon emission and cost balance analysis. Sci Total Environ.

[106] ’t Lam GP, Vermuë MH, Eppink MHM,  (2018). Multi-Product Microalgae Biorefineries: From Concept Towards Reality. Trends Biotechnol.

[107] Davis TA, Volesky B, Mucci A (2003). A review of the biochemistry of heavy metal biosorption by brown algae. Water Res.

[108] Karthikeyan S, Balasubramanian R, Iyer CSP (2007). Evaluation of the marine algae Ulva fasciata and Sargassum sp. for the biosorption of Cu(II) from aqueous solutions. Bioresour Technol.

[109] El-Sikaily A, El NA, Khaled A, Abdelwehab O (2007). Removal of toxic chromium from wastewater using green alga Ulva lactuca and its activated carbon. J Hazard Mater.

[110] Vieira MGA, Oisiovici RM, Gimenes ML, Silva MGC (2008). Biosorption of chromium(VI) using a Sargassum sp. packed-bed column. Bioresour Technol.

[111] Fagundes-Klen MR, Ferri P, Martins TD (2007). Equilibrium study of the binary mixture of cadmium-zinc ions biosorption by the Sargassum filipendula species using adsorption isotherms models and neural network. Biochem Eng J.

[112] Volesky B (2007). Biosorption and me. Water Res.

[113] Basso MC, Cerrella EG, Cukierman AL (2005). Cadmium Uptake by Lignocellulosic Materials: Effect of Lignin Content. Sep Sci Technol.

[114] Cochrane EL, Lu S, Gibb SW, Villaescusa I (2006). A comparison of low-cost biosorbents and commercial sorbents for the removal of copper from aqueous media. J Hazard Mater.

[115] European Commission (EC). Towards a Thematic Strategy for Soil Protection. Brussels. 2002.

[116] European Commission. Proposal for a Directive of the European Parliament and of the Council establishing a framework for the protection of soil and amending Directive 2004/35/EC. Brussels. 2006.

[117] USEpA. Cleanup of the Nation’s Waste Sites: Markets and Technology Trends. Washington DC; 2004.

[118] Gadd GM (2004). Microbial influence on metal mobility and application for bioremediation. Geoderma.

[119] Ross SM (1995). Toxic metals in soil-plant systems.

[120] Parthasarathy P, Narayanan S (2015). Effect of combined slow pyrolysis and steam gasification of sugarcane bagasse on hydrogen generation. Korean J Chem Eng.

[121] Shimura K, Yoshida H (2011). Heterogeneous photocatalytic hydrogen production from water and biomass derivatives. Energy Environ Sci.

[122] Cargnello M, Gasparotto A, Gombac V (2011). Photocatalytic H2 and added-value by-products-the role of metal oxide systems in their synthesis from oxygenates. Eur J Inorg Chem.

[123] Energypedia. Challenges and Issues Affecting the Exploitation of Renewable Energies in Ethiopia. Energypedia; 2020.

